# Microneedle-Based Approaches for Skin Disease Treatment

**DOI:** 10.1007/s40820-025-01662-y

**Published:** 2025-02-06

**Authors:** Yanhua Han, Xiaoyu Qin, Weisen Lin, Chen Wang, Xuanying Yin, Jiaxin Wu, Yang Chen, Xiaojia Chen, Tongkai Chen

**Affiliations:** 1 State Key Laboratory of Traditional Chinese Medicine Syndrome, Science and Technology Innovation Center, Guangzhou University of Chinese Medicine, Guangzhou, 510405 People’s Republic of China; 2 NMPA Key Laboratory for Research of Traditional Chinese Medicine Syndrome, Science and Technology Innovation Center, Guangzhou University of Chinese Medicine, Guangzhou, 510405 People’s Republic of China; 3 State Key Laboratory of Quality Research in Chinese Medicine, Institute of Chinese Medical Sciences, University of Macau, Macau, 999078 People’s Republic of China

**Keywords:** Microneedles, Androgenetic alopecia, Psoriasis, Atopic dermatitis, Hypertrophic scars, Melanoma

## Abstract

The use of microneedles (MNs) has been established as an effective transdermal drug delivery strategy that has been extensively deployed for treating various diseases, including skin diseases. MNs can surpass the constraints of conventional drug delivery methods by their superior safety and efficacy through precise targeting, while simultaneously enabling painless delivery. Currently, MNs are increasingly used as carriers for drug delivery, with the loading of insoluble drugs to improve their treatment efficiency or combining with bioactive substances for the construction of an efficient drug delivery system to maximize the effects of bioactive substances. The methods used for preparation MNs are diverse, enabling them to meet the requirements of most applications. The emergence of MNs has addressed the shortcomings associated with insoluble drugs, expanded the applications of bioactive substances, and improved their use in clinical practice. This review summarizes current information on the application of MNs in a variety of skin diseases, such as psoriasis, vitiligo, alopecia, hypertrophic scarring, atopic dermatitis, melanoma, acne, and skin infections. The current clinical applications and future opportunities for MNs in the treatment of skin diseases are also discussed. Despite substantial progress in the clinical application of MNs as delivery vectors, issues such as low drug loading and poor mechanical strength during MNs preparation remain the main challenges. Therefore, clinical implementation of MNs-based therapies remains limited, highlighting key opportunities for future research.

## Highlights

- Microneedles (MNs) are used extensively for treating skin diseases due to their capability to provide less-invasive targeted drug delivery.
- Intelligent MNs can be fabricated from biocompatible materials with specialized properties, thereby providing improved treatment efficacy.
- Currently, there are limitations in the clinical application of MNs, highlighting the significance of further investigation to facilitate the translation of this innovative technology into patient treatment contexts.

## Graphical abstract

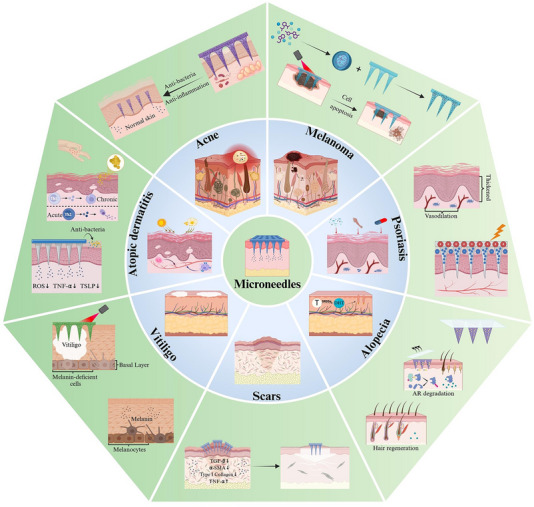

## Introduction

The skin is the largest organ in the body and represents a barrier that serves as the initial line of defense against external physical, chemical, and biological stimuli. Only 10%–20% of topically applied drugs can diffuse into the skin because of these barrier functions. Furthermore, the skin can act as a reservoir for drugs that can penetrate the skin for extended periods of time. This enables the controlled and sustained release of specific drugs with shorter biological half-lives, which would otherwise require frequent administration to maintain effective pharmacological concentrations [1]. Therefore, it is evident that the development of drug delivery systems that can enhance drug bioavailability would be advantageous, as it increases the efficacy of treatments and reduces the probability of adverse effects [2]. In recent decades, a variety of strategies have been employed to address the barrier functions of the stratum corneum (SC), the outermost layer of the epithelium. These strategies include physical and chemical methods that can enhance and regulate the transport of drugs across the skin, such as the use of chemical enhancers [3], iontophoresis [4], electroporation [5], and sonophoresis [6]. In this context, microneedles (MNs) have attracted much attention because of their novel properties, such as the potential for painless self-administration of drugs with improved efficiency and the lack of biohazardous waste production [7]. MNs are minimally invasive, making them a successful transdermal drug delivery platform. They are capable of achieving superior drug bioavailability and delivery efficiency, while improving patient compliance due to their simplicity of use and lack of associated pain [8]. MNs arrays consist of a series of needle-like structures, several micrometers in diameter, that can disrupt the outermost cutaneous layers to achieve transdermal drug delivery. The unique minimally invasive characteristics of these MNs combine with the beneficial effects of conventional intradermal injection strategies to enable the effective delivery of drugs under the skin surface [9]. MNs have been extensively investigated as tools for the delivery of a variety of chemicals and biological macromolecules, as they have the potential of being a more patient-friendly alternative to conventional drug administration techniques. Various reports have been published regarding their application in the delivery of conditioned media [10], monoclonal antibodies [11], and nanovaccines [12]. Furthermore, MNs also have wide applications in tissue engineering and regenerative medicine, such as the repair defective connective tissue and skeletal muscle, the treatment of bone-related diseases and heart disease, and the promotion of neovascularization and wound healing [13].

Skin diseases are the fourth most prevalent non-fatal medical condition, affecting an estimated one in three individuals worldwide, and are associated with a significant global socioeconomic burden [14, 15]. Alopecia, psoriasis, atopic dermatitis, vitiligo, hypertrophic scarring, melanoma, acne, and skin infections are among the most prevalent types of skin disease. There is a clear and imperative need to develop novel and more efficacious interventional approaches, as patients suffering from these skin diseases are faced with a shortage of effective options. While MNs acting on the skin can promote drug absorption [16, 17], stimulate skin repair [18], regulate immune responses [19, 20], and improve tissue structure [21, 22], they provide a research direction for the treatment of these skin diseases.

The application of MNs in a clinically relevant context, particularly for the management of skin diseases, has addressed by numerous research studies. Accordingly, this review provides a detailed overview of the most recent non-clinical research focused on the application of MNs in skin diseases. The topics discussed include the use of MNs for drug encapsulation as well as innovative applications for functional MNs, thereby expanding the potential opportunities for the treatment of skin diseases and establishing a basis for new opportunities for translational innovation. The present review offers a comprehensive examination of the potential utility of specialized biocompatible materials in the fabrication of MNs, based on the research conducted to date. This information will assist in the development of synergistic MNs-based therapeutic modalities that may facilitate more effective, targeted, and patient-friendly treatments. This review further discusses the significance of combining these MNs-based therapeutic strategies with traditional treatments to enhance their efficacy and reduce the probability of adverse effects. The results of this survey provide a comprehensive overview of the potential utility of MNs as versatile therapeutic tools that could potentially support groundbreaking advancements in the management of skin diseases in future.

## Structure of the Skin and the Development of MNs-Based Delivery Systems

**Fig. 1:**
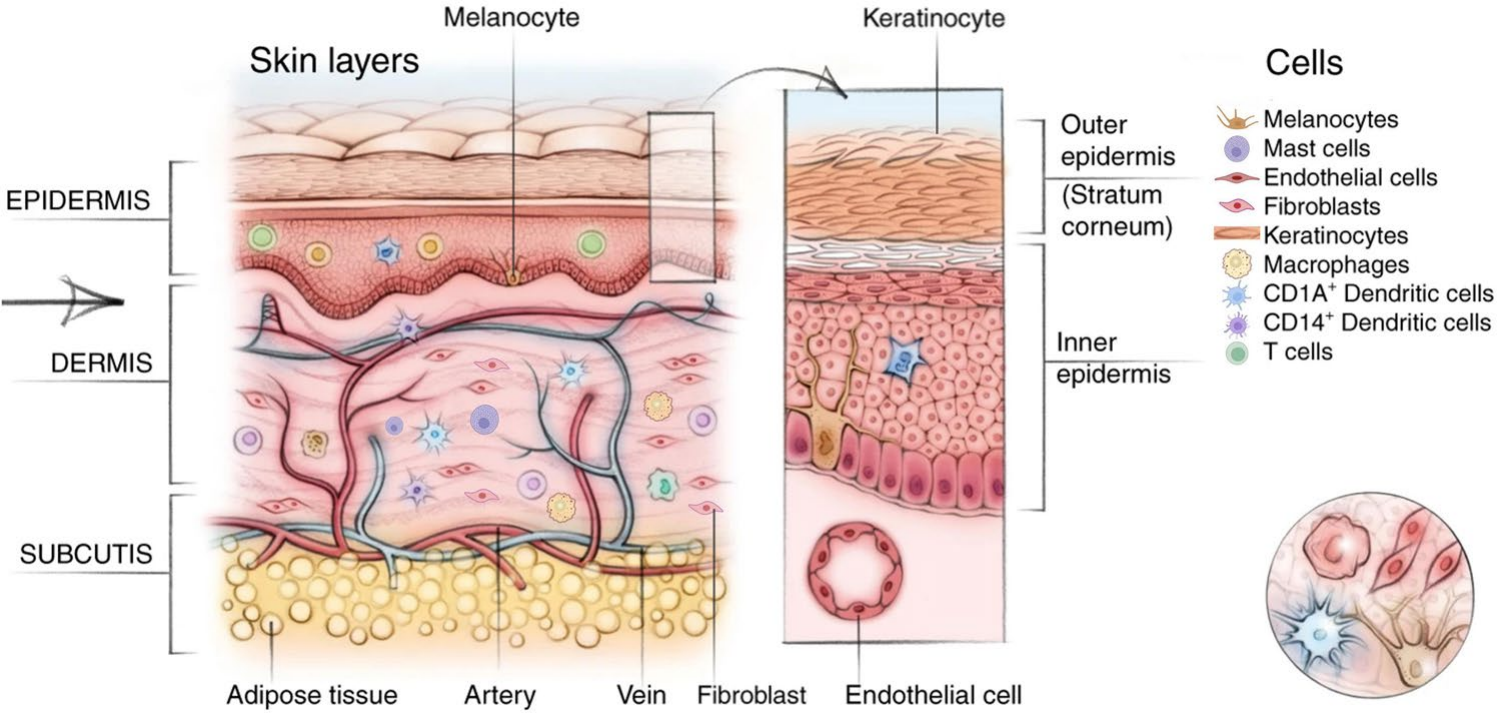
Schematic diagram of the tissue layers and cell types of the skin. Adapted from [24] with modification and permission

The skin comprises a dynamic barrier between the body and the external environment, enabling the effective regulation of body temperature and facilitating a complex range of sensations [23]. The complexity of the skin is derived from the presence of numerous tissue layers and cell types, including keratinocytes, endothelial cells, fibroblasts, melanocytes, and immune cell populations. The dense extracellular matrix (ECM) within the skin contributes to its exceptional tensile strength and flexibility [24]. The skin consists of three primary layers, namely, the epidermis, dermis, and hypodermis (Fig. 1) [24]. The epidermis is the outermost layer, about 100 μm thick in most parts of the body [25]. Epidermal stem cells are essential regulators of cutaneous regeneration and homeostasis. The dermis forms the middle layer, with a typical thickness of approximately 1 mm [26]. It includes various dermal cells and lies between the epidermis and the subcutaneous fat layer [27]. The dermis contains extensive elastin and collagen protein networks that support the flexibility and mechanical strength of the skin. The hypodermis is the softest layer and has a thickness of several millimeters [28]. Subcutaneous fat functions as a site for energy storage side, as well as providing insulation and padding. The epidermis is capable of generating appendages [29], in the form of hair follicles (HFs) and associated sebaceous glands in certain areas of the body. The length and thickness of the hair can vary, with long terminal hairs and finer, shorter vellus hairs [30], serving as an additional protective barrier against external environmental factors [31]. Therefore, the skin relies on a single layer of proliferating cells that ultimately produces terminally differentiated stratified layers. These layers are continually sloughed off from the skin surface and are replaced by the outward movement of the underlying cells. The epithelium and the dermis are separated by an ECM and tyrosine growth factor (TGF)-rich basement membrane that stimulates the proliferation of the innermost epidermal basal layer [32]. The distinct physiological and structural attributes of the skin present a favorable prospect for the delivery of therapeutic substances through the skin to treat a range of diseases, owing to the presence of numerous lymphatic vessels and blood vessels within the skin that interconnected with the rest of the body [30]. The skin not only acts as a barrier against pathogens, but it also effectively limits the transdermal permeation of drugs.

MNs have attracted significant interest as a tunable topical delivery platform for a diverse array of drug cargos due to their minimally invasive and user-friendly administration [33]. The short length of MNs ensures that they do not reach the capillaries, resulting in minimal discomfort and a lack of bleeding. They typically range in length from ten to over 1000 µm [34]. The administration of various kinds of drugs can be facilitated by the MNs through reversible microchannels, resulting in less pain compared with traditional hypodermic needle-based delivery [35]. The specific structure of the MNs enables their ready penetration of the *SC* allowing entry to the viable epidermis [36].

**Fig. 2:**
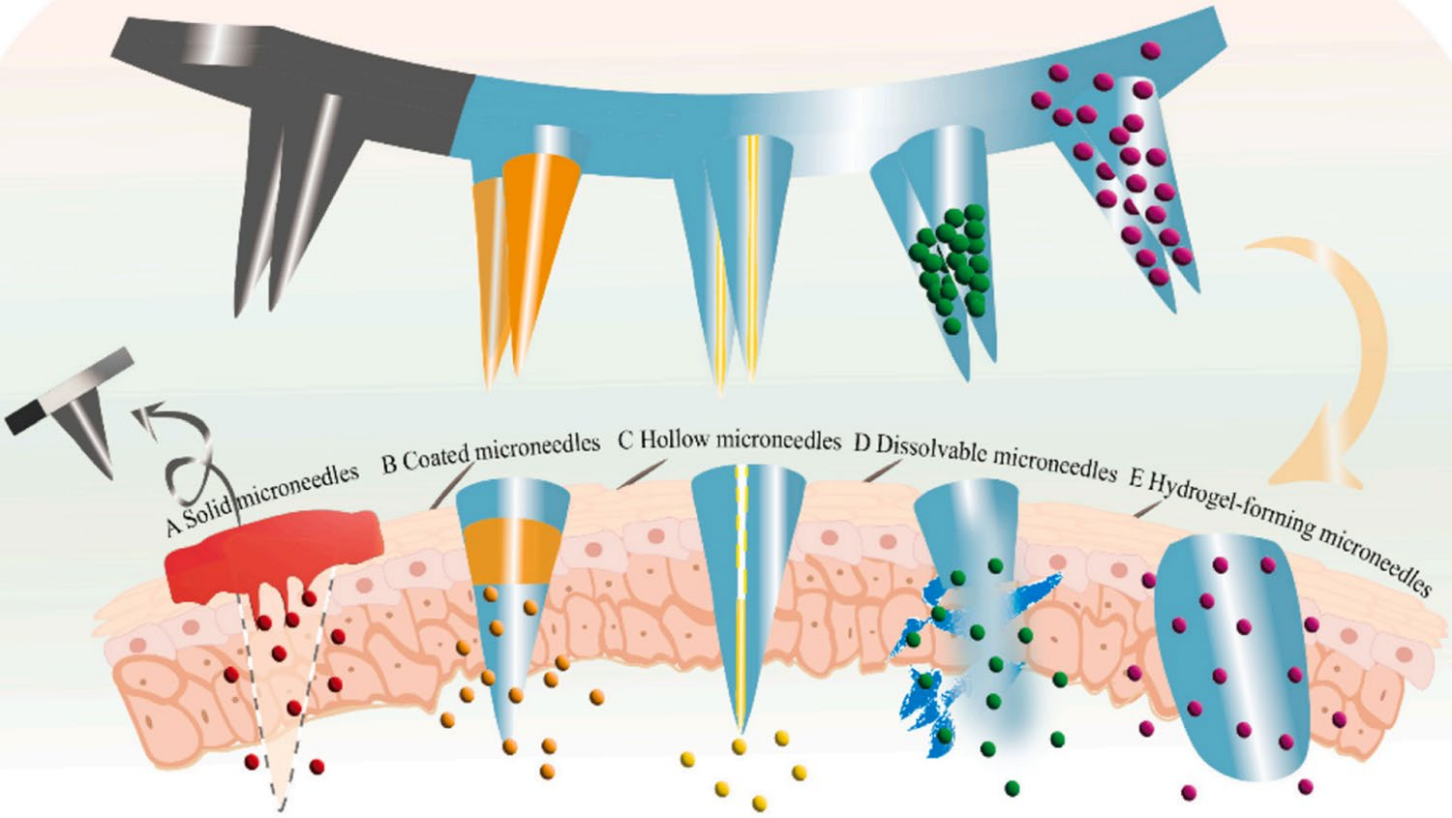
The process of drug delivery by various types of MNs. **A** Solid MNs: after application to the skin, they can pierce the stratum corneum and form micron-sized channels. Removal of the MNs allows the transdermal release of drugs applied on the skin surface; **B** Coated MNs: the drugs are coated on the MNs surface. As the MNs penetrate the skin, the outer drug coating enters the skin and is removed when the drug release is completed; **C** HMNs: these are characterized by a channel in the center of the MNs, through which the drug solution can flow into the skin; **D** DMNs: the drugs are mixed with polymer materials to form individual needle tips. When the MNs are inserted into the skin, the drugs are released as the polymer materials dissolve. On completion of the drug release, the base of the remaining MNs is removed; **E** Hydrogel-forming MNs: Hydrogel-forming MNs have a three-dimensional network structure, can absorb tissue fluid, and expand after insertion into the skin. The drugs within the structure are released due to the concentration gradient. On completion of the drug release, the MNs patch can be completely removed. Adapted from [37] with permission. MNs: microneedles; HMNs: hollow microneedles; DMNs: dissolvable microneedles

Five major classes of MNs have been designed for transdermal drug delivery. These include solid MNs, coated MNs, hollow MNs (HMNs), dissolving MNs (DMNs), and hydrogel-forming MNs (Fig. 2) [7, 37]. Solid MNs, after application to the skin, can penetrate the SC and form micron-sized channels. After the MNs are removed, transdermal release of the drug occurs on the skin surface (Fig. 2A). Solid MNs are typically composed of materials such as metal, silicon, titanium, or glass, which facilitate the movement of materials through the SC. Nevertheless, the use of metal or silicon-based solid MNs on the skin may pose challenges due to their relatively poor biocompatibility and the potential harm caused by broken MNs made of these materials [38]. Coated MNs involve coating the drug onto MNs followed by insertion into the epidermis for subsequent dissolution of the drug coating (Fig. 2B). Furthermore, it is also possible to increase the long-term stability of MNs by coating them with drugs in a solid phase [39]. The function of HMNs is analogous to that of miniaturized arrays of traditional syringes, enabling the use of an external syringe pump to administer drugs after piercing the skin (Fig. 2C). This allows the volume of MNs-mediated drug delivery to be increased to the highest level of skin tolerance [40]. The production of DMNs involves the combination of drugs and biodegradable polymers, which are then cured to produce a product that possesses specific mechanical properties when it permeates the epidermis [41]. The US Food and Drug Administration (FDA) has approved the use of the natural polysaccharide hyaluronic acid as a dermal filler, and it is extensively employed in the development of DMNs due to its superior clinical safety and satisfactory mechanical strength [10]. Furthermore, recent research indicates that DMN patches made with sulfobutyl ether-β-CD (SCD) as the matrix have not only strong mechanical properties and an ultra-fast dissolution rate but can also load both hydrophilic and hydrophobic drugs [42]. When DMNs penetrate the epidermis, the polymer is dissolved by the fluid in the underlying tissue, leading to the release of the drug and thus providing high local concentrations of the loaded drugs in the target site (Fig. 2D). The hydrogel-forming MNs are produced from hydrogel polymers, colloids, or proteins. They can swell within the skin by absorbing fluids from the tissue to form porous aqueous micro conduits. They are capable of resisting the sealing of skin pores and allowing for their complete removal from the tissue without any associated residue [43]. The density of hydrogel fiber cross-linking can also be adjusted to regulate drug release rates (Fig. 2E) [44]. The potential for developing MNs systems with distinct release rates is available through the selection of a variety of polymeric materials with unique properties, providing further possibilities for MNs development.

MNs are well-suited for the therapy of dermatological diseases due to their numerous advantageous properties. Firstly, the tips of MNs are retained within the skin to facilitate the sustained release of drugs, thereby improving their skin retention [9]. Secondly, MNs can readily pierce the dermis, allowing for the efficient delivery of payloads of interest, regardless of their molecular weight [45]. Thirdly, the structural characteristics of MNs facilitate their interaction with an extensive range of drugs, including molecules of varying sizes. These MNs can readily penetrate the SC to achieve the subcutaneous delivery of chemotherapeutic drugs and photothermal agents for tumor targeting, for example, by providing a means of improving overall therapeutic efficiency while minimizing undesirable side effects [46]. MNs enable larger molecules to penetrate the SC, thereby achieving a higher level of bioavailability than that achieved through oral administration [41]. The use of MNs to disrupt the ordered lipid bilayer structure of the SC has been the subject of an increasing number of recent studies. This technique has the potential to generate microchannels that enable the painless and reversible delivery of drugs into the skin [47], making it an ideal means for treating skin diseases [48].

## Applications of MNs to Treat Androgenetic Alopecia

Androgenetic alopecia (AGA) is the most common cause of alopecia worldwide, and it has an adverse effect on the self-esteem and quality of life of patients. Therefore, there is a need for effective treatments for this condition [49], with affected individuals exhibiting progressive hair thinning [10]. The pathophysiology of AGA is multifactorial, with the 5α-reductase-mediated conversion of testosterone into dihydrotestosterone (DHT) playing a central role in this context. This is due to the greater affinity of DHT for androgen receptors (AR) than testosterone [50]. This is believed to contribute to the progressive miniaturization and eventual loss of HFs by modulation of the HF development cycle when DHT binds to AR expressed in HFs, particularly those in areas of the scalp that are sensitive to AGA, namely, the crown and temples [33]. Furthermore, AGA is also influenced by the hair follicle growth cycle. The hair follicle growth cycle is usually divided into three phases: anagen, catagen, and telogen. In patients with AGA, the hair follicle growth cycle is disrupted, manifesting largely as shortened anagen and an extended telogen phases of the hair follicles [37]. Currently, the two primary strategies for the management of alopecia are surgery and drug treatment. The transplantation of HFs is restricted by high costs, a scarcity of suitable donors, and low transplant survival rates. First-line drugs used to treat patients include oral finasteride and topical minoxidil [10, 49]. Finasteride and minoxidil treat AGA by the competitive inhibition of type II 5α-reductase and improving local blood circulation, respectively [51, 52]. Studies have also suggested regulation of the hair follicle growth cycle through the Wnt/β-catenin signal and the Shh/Gli signaling to treat hair loss [37, 53–55]. Furthermore, the application of MNs in the treatment of AGA has been extensively studied. The mechanical stimulation provided by MNs promotes hair regeneration. On the other hand, MNs serve as a drug delivery system to improve drug targeting toward HFs and enhance effectiveness.

Multiple attempts have been made to use MNs as a device to stimulate hair regrowth [37]. The applications of MNs in AGA are shown in Table 1. MNs can facilitate the minimally invasive delivery of drugs by combining the advantages of intradermal injections and the convenience of transdermal approaches [43]. Hyaluronic acid (HA) can protect against chemotherapy-induced hair loss by increasing cell–cell adhesion and reducing the cell substratum, thereby enhancing the proliferative, migratory, and aggregative activities of human dermal papilla (HDP) cells. Additionally, it can influence the development of keratinocytes. HA can also provide a suitable environment for optimal functioning of HDP cells [50]. There is an increasing interest in the application of MNs for delivery of drugs or other bioactive compounds to promote hair regeneration.

**Table 1:** Application of MNs in androgenetic alopecia

Types	Active substances	Type of MNs	Function	Limitations	References
PRP-MNs	PRP	DMNs	PRP-MNs provide a painless, minimally invasive, and sustainable effect of PRP for hair regrowth	The bioactivity of GFs in platelets was maintained for only 6 h when PRP was prepared under conventional conditions	[59]
PROTAC-MNs	Androgen Receptor-PROTAC	DMNs	The PROTAC-MNs showed high biocompatibility, one-step administration, and long-lasting efficacy, without causing systemic toxicity or androgen deficiency-related disorder in vivo	Although PROTAC-MNs have shown superior hair regrowth ability in the AGA recurrence mouse model, the exact mechanism underlying them has not been fully elucidated	[49]
DMN-VPA	Valproic acid	DMNs	DMN-VPA maximized VPA delivery efficacy while promoting HF regrowth via microwounding of the skin	There remains a need for future detailed studies on the effects of microwounding depth, distance between microwounds, and extended animal tests focusing on microwounding	[56]
MnMNP	MnPS_3_	DMNs	MnMNPs demonstrated a superior hair regeneration capability at a reduced application frequency and provided a general framework for the identification of SOD-like nanozymes through ML techniques	–	[60]
MXD + HA-MN	MXD, HA	DMNs	MXD + HA-MN released MXD as a controlled drug delivery system, thereby extending its widespread application	The loading of MXD in MNs patch is relatively low	[50]
CM-MNs	CM from hypoxia-pretreated MSCs	DMNs	Pro-angiogenic factors can be delivered directly into the epidermis by CM-MNs in a single, minimally invasive step by puncturing the SC	The specific factor involved and its corresponding role need further investigation	[10]
HMN	MSC-derived exosomes, UK5099	HMNs	The HMN facilitated the transport of loaded cargoes directly into the HFSC niche in a painless and minimally invasive manner, thus augmenting the transdermal efficiency with reduced drug dosages	–	[61]
FIN-NLC-MN	FIN	DMNs	The FIN-NLC-MN markedly increased the therapeutic efficacy and skin retention of FIN	The delivery of DMNs to hairy skin remains an obstacle for AGA treatment	[33]

PRP, platelet-rich plasma; MNS, microneedles; PRP-MNS, platelet-rich plasma microneedles; GFs, growth factors; PROTAC-MNs, proteolysis-targeting chimera-microneedles; AGA, androgenetic alopecia; MnMNP, MnPS_3_ microneedle patch; MXD, Minoxidil; HA MN, hyaluronic acid; VPA, valproic acid; ML, machine learning; CM-MNs, conditioned media-microneedles; SC, stratum corneum; MSC, mesenchymal stem cell; HFSC, hair follicle stem cell; FIN-NLC-MN, finasteride-loaded nanostructured lipid carriers-microneedles; FIN, finasteride; NLC, lipid nanocarriers

**Fig. 3:**
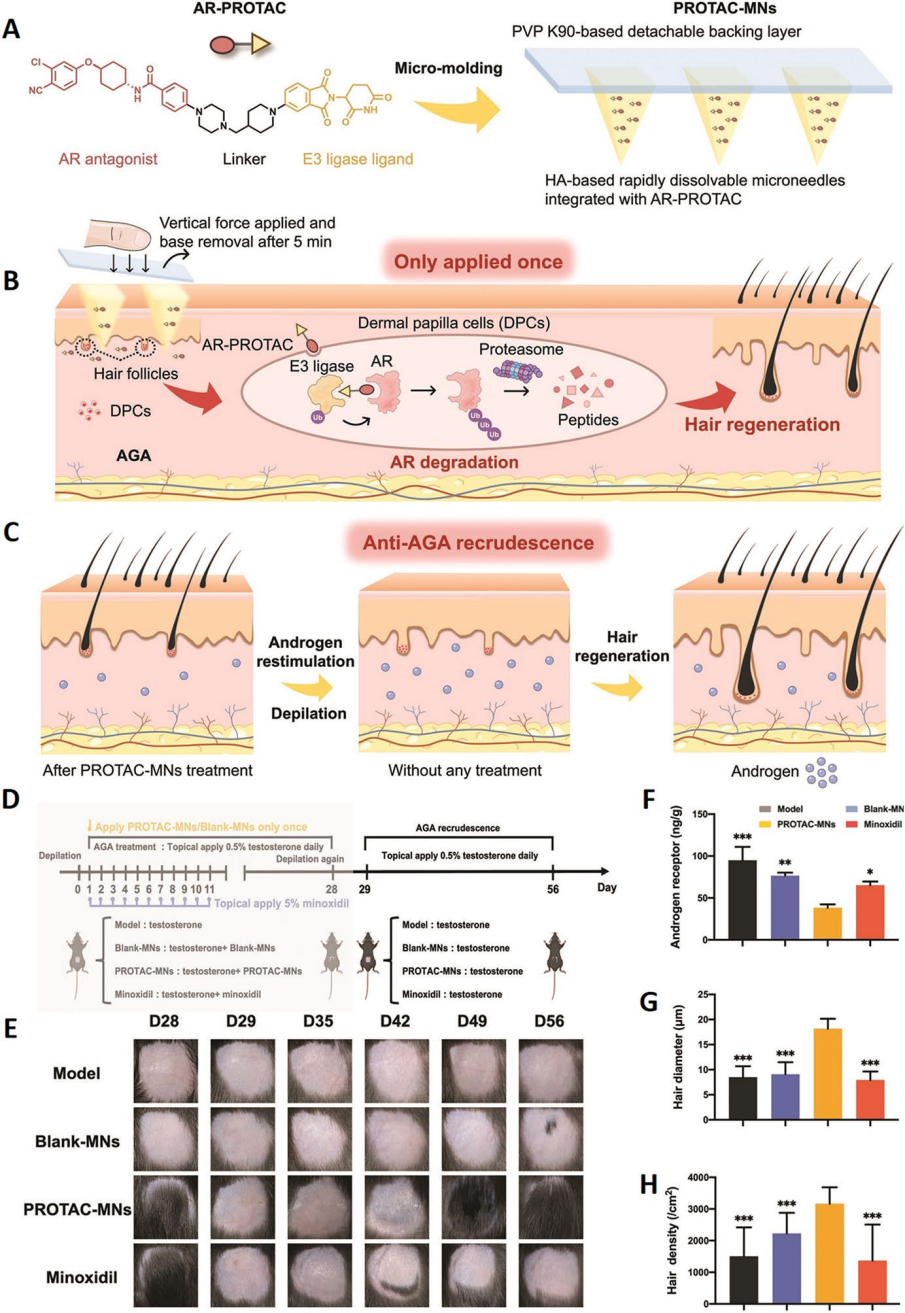
Schematic overview of PROTAC-MNs-based AGA treatment. **A** PROTAC-MNs synthesis approach. AR-PROTACs were synthesized and encapsulated within HA-based MNs, with the prepared patch consisting of a PVP-K90 backing layer. **B** The backing could be removed 5 min after the PROTAC-MNs were pressed into the skin using vertical force applied with the forefinger, allowing the needles to penetrate the skin and dissolve upon contact with the fluid within the underlying tissue. The constructed AR-PROTAC was thus delivered successfully to HF-containing regions, thereby facilitating the degradation of AR within dermal papilla cells (DPCs) in the HFs through the ubiquitin–proteasome system. PROTAC-MNs were capable of effectively promoting hair regrowth as a treatment for AGA after a single application. **C** PROTAC-MNs also showed promise with respect to their anti-AGA recurrence effects following a single round of administration in the previous hair cycle. **D** AGA recurrence model was established by the topical application of testosterone for a further consecutive 28 days after the redepilation of the regrown hair in AGA-treated mice, without any treatment in all groups. **E** Representative images of the depilated regions on days 28, 29, 35, 42, 49, and 56 post-first-depilation in each group. **F** Quantification of ARs in the treated-skin on day 56 post-first-depilation (*n* = 3). **G** Diameters of the regenerated hairs on day 56 post-first-depilation (*n* = 20). **H** Quantification of hair densities on day 56 post-first-depilation (*n* = 10). *p < 0.05; **p < 0.01; and ***p < 0.001 versus the PROTAC-MNs group. Adapted from [49] with permission

Minoxidil (MXD) and finasteride, both of which are currently FDA-approved drugs for AGA, have made substantial progress in the treatment of AGA. However, long-term use of MXD and finasteride can lead to side effects such as skin itching, redness, decreased libido, depression, and ejaculation disorders [37]. This progress has been further developed by the use of MNs-loaded drugs in a drug delivery system [7, 50]. AR-targeting PROTAC (proteolysis-targeting chimera) constructs have also demonstrated potential by modifying AR activity to eliminate the adverse effects of DHT on HFs. This is due to the essential role played by androgen binding to ARs in the pathogenesis of AGA. An AR-PROTAC (TJA-107) construct has been successfully synthesized and integrated with HA-based MNs and a polyvinylpyrrolidone (PVP)-k90-based detachable backing layer to form a PROTAC-MNs patch (Fig. 3A) [49]. The PROTAC-MNs patch enables effective delivery of the encapsulated AR-PROTAC to the HFs-associated regions through its micropunctures, and also utilizes the ubiquitin–proteasome system to degrade the ARs in the DPs to promote hair regrowth (Fig. 3B). In the preclinical study of AGA treatment, PROTAC-MNs showed significant efficacy. After successful modeling of AGA and administration of an initial dose of PROTAC-MNs, faster transition of HFs into the anagen phase was observed, together with a significant increase in Ki67 expression, with similar hair quality compared to the routine administration of minoxidil. Meanwhile, an in vivo assessment in mice on day 28 indicated that the expression levels of ARs in the depilated skin of the PROTAC-MNs group was significantly reduced. PROTAC-MNs have also shown the potential to reduce recurrence rates in subsequent AGA recurrence models (Fig. 3C). On the 28th day, after successful modeling of AGA, all mice were dehaired and treatment with testosterone was continued to model AGA recurrence (Fig. 3D). In contrast to the high rate of recurrence associated with minoxidil, the PROTAC-MNs were administered only on the first day of the AGA modeling, yet hair regeneration was still achieved at the subsequent 29–56-day AGA recurrence model (Fig. 3E). After successfully creating the AGA model and giving the first dose of PROTAC-MNs, the more rapid transition of HFs to the anagen phase was observed, together with significant increases in the diameter and density of the hair, demonstrating similar hair quality compared to the routine administration of minoxidil. Meanwhile, in the in vivo assessment on day 56, the expression levels of ARs in the depilated skin of the PROTAC-MNs group were significantly reduced (Fig. 3F–H). This demonstrates the anti-AGA recurrence ability of AR-PROTAC to reduce AGA recurrence. Valproic acid (VPA) has also been developed as a potential treatment for AGA and can facilitate wound re-epithelialization, potentially providing a more general usefulness for the regeneration and maintenance of HFs [56]. Furthermore, the integration of these innovative treatments into clinical regimens may contribute to improved clinical outcomes for AGA patients with AGA, thereby enabling individuals to more effectively manage and overcome this prevalent and distressing condition.

**Fig. 4:**
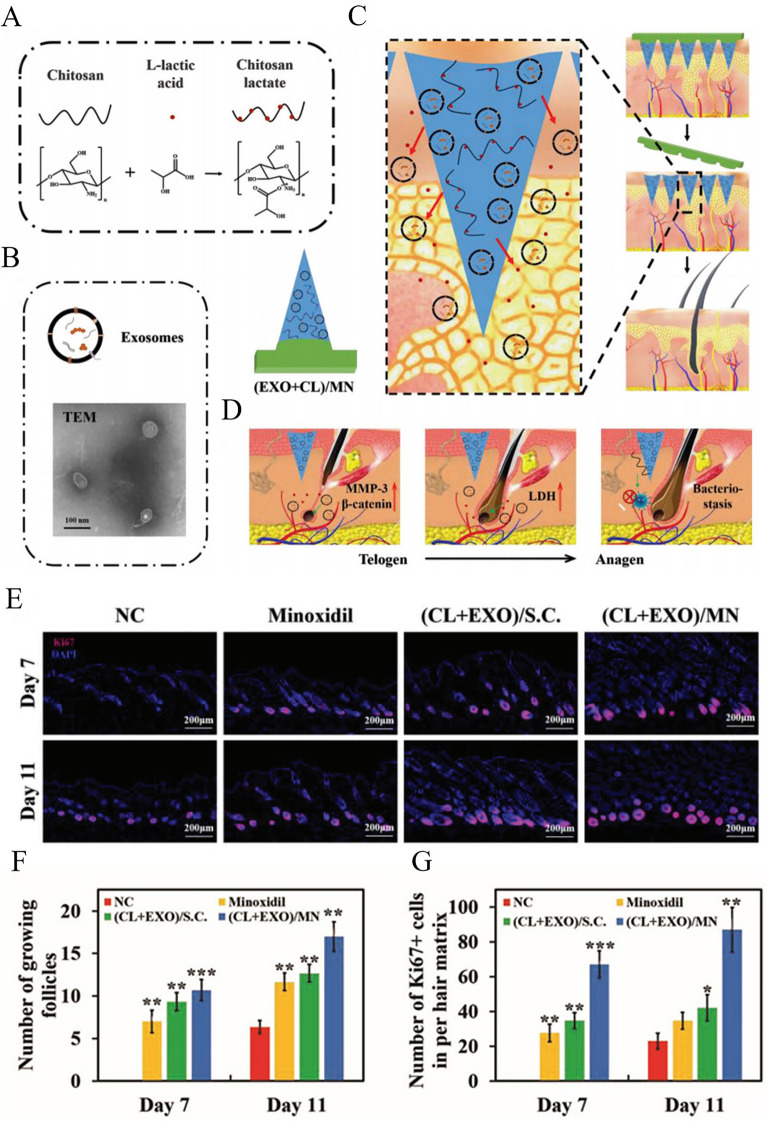
Schematic overview of the therapeutic application of (EXO + CL)/MNs for hair regeneration. **A** Prepared chitosan lactate (CL) and **B** extracted exosomes (EXO) were loaded into needle tips to develop (EXO + CL)/MNs preparations. **C** HA component of the prepared MNs dissolved rapidly after insertion into the skin, resulting in the polyvinyl alcohol (PVA) tips of the needles remaining within the skin. **D** CL-derived L-lactic acid was able to promote dermal papilla cell (DPC) lactate dehydrogenase (LDH) activity, while EXO-mediated β-catenin and MMP-3 upregulation was also observed in DPCs, synergistically leading to activation of anagen in HFs and the promotion of hair regeneration. The antibacterial effects of CL were also sufficient to protect against bacterial infections caused by MNs insertion. **E** Immunofluorescence microscopy of Ki67 on days 7 and 11 after administration (scale bar = 200 μm). **F** Quantitative analysis of the number of hair follicles shown in Fig. 4E. **G** Quantitative analysis of the number of Ki67-positive cells shown in Fig. 4E. NC: negative control. Data are expressed as the mean ± SD (*n* = 6), and *p*-values are calculated using one-way ANOVA with Bonferroni correction, *p < 0.05; **p < 0.01; ***p < 0.001. Adapted from [45] with permission

Efforts to treat AGA have recently moved away from the reliance on traditional pharmaceuticals and toward exploring a wider variety of bioactive compounds. The use of conditioned media (CM) derived from mesenchymal stem cells (MSCs) has been a particularly promising research avenue. These cells secrete a diverse array of cytokines and growth factors that can stimulate angiogenesis in the vicinity of HFs [10]. Adipose-derived stem cell exosomes (EXO) have also been explored in this therapeutic context [6, 45]. The dermal papilla is essential for the cycling of these structures and serves as the primary focus of HF signaling. Hair regeneration can be effectively accomplished by facilitating the entry into anagen in these cells through the accelerated rate of DPC proliferation. A study successfully synthesized chitosan lactate (CL) using chitosan and l-lactic acid as raw materials (Fig. 4A), and loaded CL and adipose-derived stem cell EXO into a separable patch of MNs for inducing hair regeneration (Fig. 4B) [45]. After the insertion of (EXO + CL)/MNs into the skin, the needle tip remained in the skin (Fig. 4C). EXO was then continuously released from the needle tip and absorbed by DPCs, thereby inducing increased expression of matrix metallopeptidase 3 (MMP3) and β-catenin [45]. MMP3 can activate the Wnt/β-catenin signaling pathway by antagonizing the function of Wnt5b, thereby promoting hair growth [57, 58]. In addition, L-lactic acid derived from CL can stimulate lactate dehydrogenase, thereby increasing cell proliferation (Fig. 4D). This regulation of regenerative activity led to the finding of Ki67-positive cells in the HFs of all treatment groups on the 7th day (Fig. 4E), and (CL + EXO)/MN significantly activated more HFs and Ki67-positive cells than the topical application of minoxidil, and the activated hair follicles and Ki67-positive cells on the 11th day were 1.5 times and 2.5 times more abundant, respectively, than those after treatment with topical minoxidil (Fig. 4F, G). The extraction of beneficial substances with the capability to promote hair regeneration and their loading into MNs can provide mechanical properties sufficient to deliver these substances to target HFs, thereby driving accelerated hair regeneration more effective than that achieved by the first-line treatment of topical minoxidil. These novel therapeutic approaches are an important advance toward the development of more effective targeted regenerative strategies, and they represent a dramatic change in the treatment of AGA. Although these treatments are currently experimental and are still in the early stages of development, they have the potential to significantly transform the field of AGA treatment space. Further clinical trials and research endeavors to optimize the safety and efficacy of these methods are essential, as they will offer more personalized and effective treatments for individuals with AGA.

## Applications of MNs to Treat Psoriasis

Psoriasis is a severe non-communicable disease, classified as a chronic inflammatory systemic disease, that predominantly affects the skin and joints [62]. Infiltration of the epidermal by inflammatory cells and keratinocyte hyperproliferation result in the formation of thick, scaly, red plaques, which are considered the cutaneous manifestations of this skin disorder [11]. Psoriasis has an adverse effect on the patient’s quality of life and is associated with a variety of metabolic, cardiovascular, and arthritic comorbidities, affecting more than 100 million individuals worldwide [20]. The treatment of psoriasis continues to be a persistent clinical challenge due to the highly variable and unstable nature of therapeutic efficacy [63]. Recent advances in MNs-based dermal drug delivery strategies, however, have provided new opportunities for efficacious treatment. The applications of MNs in the treatment of psoriasis are shown in Table 2. Indeed, numerous studies have used MNs to treat psoriasis in a minimally invasive and painless manner by penetrating the upper layers of the epidermis [41, 64].

**Table 2:** Applications of MNs in psoriasis

Types	Types of MNs	Function	Limitations	References
HMNs of the dual-molding process	HMNs	HMNs decreased the production cost and reduced the dosage, and associated side effects of drugs	–	[41]
mD-MN	Coated MNs	This wearable mD-eMN system has been demonstrated to be an effective and versatile platform for treating psoriasis. It combines flexible electronics and chemical medications, allowing for the use of electrical stimulation and chemical therapeutics in the treatment of various diseases	Unable to cover large-area lesions	[69]
MTX-loaded MNs	DMNs	MTX-loaded MNs decreased the side effects associated with injection or oral administration and enhanced the skin penetration of the drug	The mechanical strength of MTX-loaded MNs is limited by the concentration of MTX	[70]
MXene HA MNs-loaded IL-17 mAbs	DMNs	The combination of MNs and emerging biologics is advantageous for the sustained release and drug targeting of biologics, and it has proposed a strategy for the clinical transformation of biologics	–	[11]
CA-EV-loaded MNAs	DMNs	CA-EV-loaded MNAs improved the drug stability and solubility, thereby providing a strategy for the local application of other insoluble drugs	The loading amount of curcumin in EVs is low	[64]
TAMN	DMNs	TAMN expands the range of drugs that DMN can carry and extends the application of supramolecular materials in the treatment of various skin diseases	The loading amount of triamcinolone acetonide (TA) is low	[71]
MTX@HMSN/CS-loaded MNs	DMNs	MTX@HMSN/CS-loaded MNs are a promising strategy for the treatment of psoriasis in alleviating psoriatic-like skin inflammation due to their durability, effectiveness, convenience, and safety	High drug loading leads to a decrease in the mechanical strength of MNs	[72]
MTX/EGCG-HP gel MNs	Hydrogel-forming MNs	The MN patches rapidly released MTX in a diffusive manner and sustained the release of EGCG in an H_2_O_2_-responsive form	–	[68]

HMNs, hollow microneedles; MTX, methotrexate; MTX-loaded MNs, methotrexate-loaded microneedles; mD-MN, multi-component drug-loaded microneedle; EGCG, epigallocatechin-3-gallate; DMNs, dissolvable microneedles; HA, hyaluronic acid; MXene HA MNs-loaded IL-17 mAbs, MXene hyaluronic acid-loaded IL-17 monoclonal antibodies; CA-EV-loaded MNAs, curcumin albumin-encapsulated extracellular vesicles-loaded microneedle arrays; CA, curcumin albumin; EV, extracellular vesicle; TAMN, TA-loaded hydroxypropyl β-cyclodextrin dissolvable microneedle; CS, chitosan; HMSN, hollow mesoporous silica nanoparticles; MTX@HMSN/CS-loaded MNs, methotrexate-loaded chitosan-coated hollow mesoporous silica nanoparticles-loaded microneedles; MTX/EGCG-HP gel MNs, cross-linked polymer gel microneedles containing methotrexate, epigallocatechin-3-gallate, and phenylboronic acid-modified hyaluronic acid (HP)

**Fig. 5:**
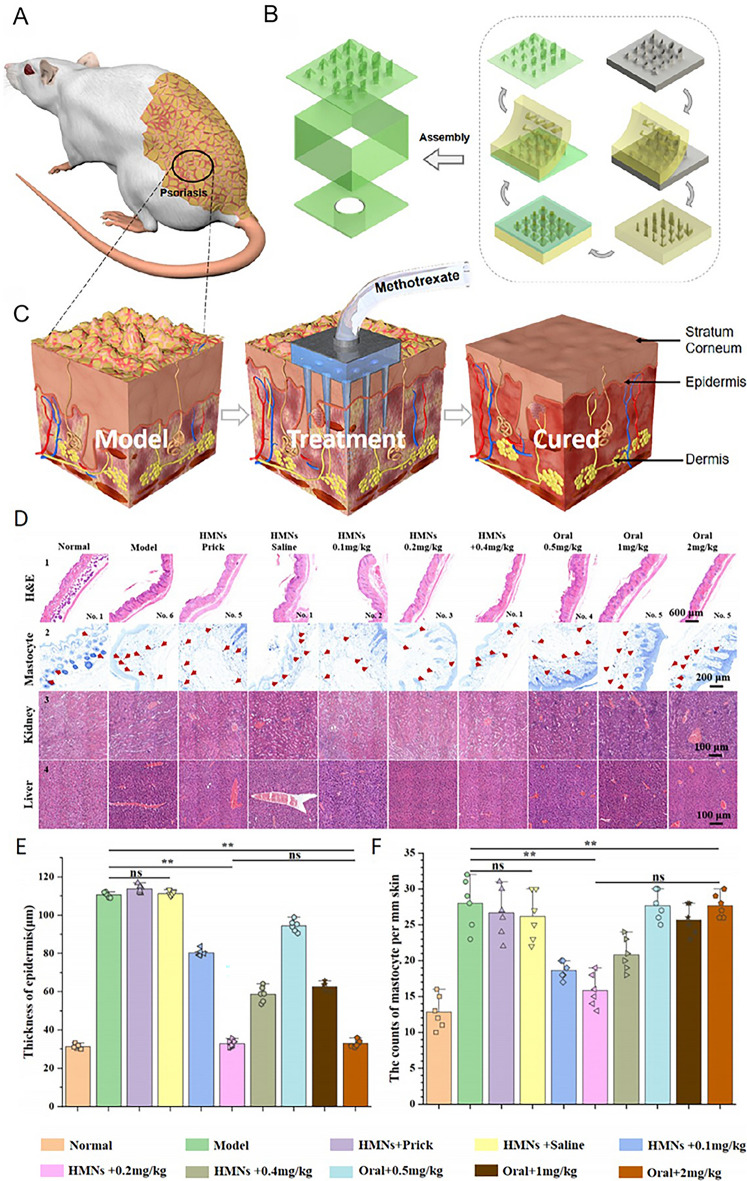
HMNs fabrication using a dual-molding process for in vivo use. **A** Mouse with psoriasis. **B** 3D structure of the skin during treatment with HMs. **C** Structural overview of the process of HMNs treatment. **D** Analysis of H&E staining (scale bar = 600 μm), mastocyte counts (scale bar = 200 μm), and kidney and liver morphology (scale bar = 100 μm). **E** Epidermal thickness analysis (*n* = 6). **F** Mastocyte counts (n = 6). No significance (ns), *p < 0.05; **p < 0.01; and ***p < 0.001. Adapted from [41] with permission

The use of methotrexate, a first-line drug for the treatment of psoriasis, with MNs can enhance its curative effects by overcoming its poor water solubility and thus offering a novel strategy for the treatment of psoriasis. Numerous studies have investigated the potential of MNs in the treatment of psoriasis, with an emphasis on both drug loading [62], and structural modification of the MNs [65]. These treatment strategies aim to optimize direct drug delivery to psoriasis-affected sites, thereby minimizing the likelihood of systemic adverse effects and maximizing therapeutic efficacy. Researchers have explored the use of a dual-molding process to achieve highly precise 3D-printed HMNs fabrication using a light-curing resin approach to treat psoriasis in mice [41]. Precision 3D-printed HMNs composed of patches and microchambers were found to minimize drug-related toxicity and side effects and were sufficient to achieve comparable efficacy at a drug dose of 0.1 times the oral dose (Fig. 5A–C). Hematoxylin and eosin (H&E) staining, mammary gland cell counts, and kidney and liver sampling were performed on mice after euthanasia. H&E staining was used to assess the epidermal thickness (Fig. 5D_1_, E). It can be seen that the results of HMNs + 0.2 mg kg^−1^ and oral + 2 mg kg^−1^ were similar to those of the normal group. Mammary gland cell counts (Fig. 5D_2_, F) were used to indicate skin allergy. The results of HMNs + 0.2 mg kg^−1^ were similar to those of the normal group, indicating less allergic reaction. Analysis of the kidneys and liver (Fig. 5D_3_, D_4_) showed that the number of blood vessels increased in all groups except the normal group and the HMNs + 0.1, 0.2, and 0.4 mg kg^−1^ groups, which also indirectly suggested greater liver and kidney damage. This MNs-based treatment strategy is highly innovative and adaptable, allowing for the further optimization of drug concentrations to enhance the safety and increase the scope of clinical applications.

Furthermore, several studies have reported the integration of MNs with traditional Chinese medicine (TCM) methods for the treatment of psoriasis [47], enhancing the efficacy of these medicinal compounds while also broadening the potential applications of TCM, thus advancing psoriasis management as well as TCM practices in general.

Innovative therapeutic strategies for psoriasis have emerged in recent years, demonstrating the increasing interest in the implementation of personalized and targeted approaches. Psoriasis is characterized by immune hypersensitivity, indicative of an autoimmune disorder [66]. Therefore, the objective of regulatory T cell (Treg)-based therapies is to suppress these responses using natural regulatory processes [17, 20]. Efforts to load MNs with gene-editing agents hold significant promise, as they have the potential to directly target the genes underlying the pathogenesis of psoriasis and regulate the root cause of the disease at the cellular level, thereby achieving highly specific treatment. Furthermore, studies have shown that nucleic acid delivery can exert good therapeutic effects in the treatment of psoriasis. However, these nucleic acid drugs are usually used topically on the skin and are required to reach the inner layer of the skin [67]. Therefore, loading nucleic acid drugs on MNs is important. There are also ongoing efforts exploring the design of intelligent MNs-based drug delivery platforms that are responsive to particular stimuli within the cutaneous microenvironment, thereby enabling the controlled release of pharmaceuticals [11, 68]. The combination of multiple therapeutic agents in a single MNs patch can further optimize these strategies, providing the benefits of a reduced risk of adverse systemic effects, a more reliable therapeutic efficacy, and a lower frequency of drug administration.

**Fig. 6:**
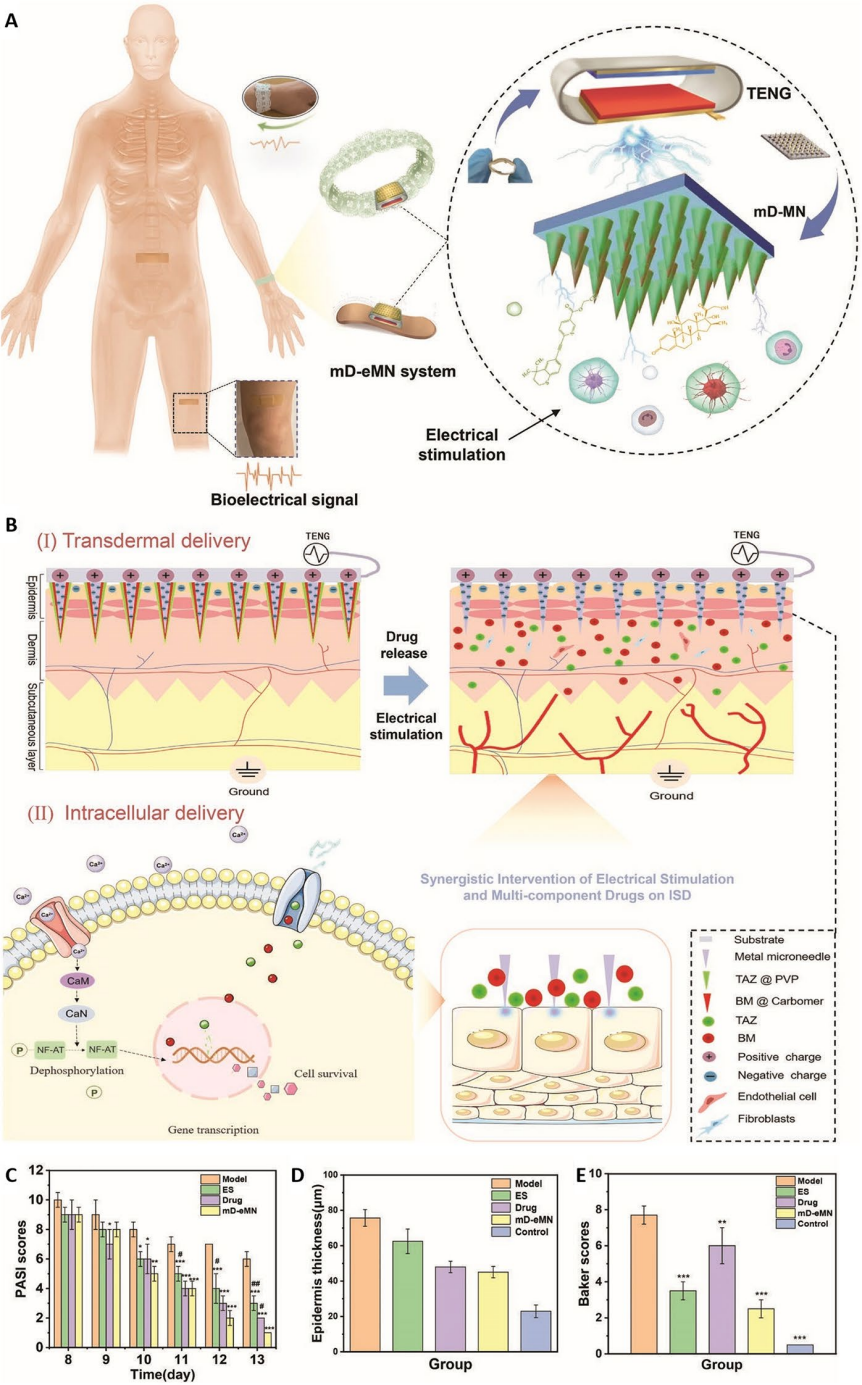
Development and application of an mD-eMN system for the accelerated regeneration of the skin in inflammatory skin diseases. **A** Structural overview of the mD-eMN system, which consists of mD-MN and a TENG that can be readily worn by humans, generating physiological electrical stimulation capable of promoting drug absorption and the restoration of tissue homeostasis. **B** Schematic overview of the transdermal and intracellular delivery achieved with the mD-eMN system: (I) This system consists of a bandage or bracelet attached to the inflamed skin capable of automatically generating pulsed electrons at the tips of the mD-eMN, thereby mediating enhanced multi-component drug absorption through (II) transient cell membrane pathways and the activation of Ca^2+^ signal transduction pathways, thus accelerating the restoration of tissue homeostasis and the alleviation of inflammation. **C** PASI scores, **D** epidermal thickness, and **E** Baker scores of psoriatic skin lesions treated with different treatments. Adapted from [69] with permission

Pulsed electrons can promote drug absorption by activating electroactive cells in the skin and have the potential to facilitate the reconstruction of homeostasis in the skin tissue. Intelligent MNs have been developed to further improve this strategy to reduce psoriasis-related skin inflammation. To achieve this objective, researchers have incorporated metal MNs containing multi-component drugs, thereby constructing a wearable and self-powered multi-component drug-loaded electronic MNs (mD-eMNs) system that employs triboelectric nanogenerators (TENGs) (Fig. 6A) [69]. Under physiological movement, TENGs can generate pulsed electrons that act directly on the tissue, achieving an electroporation effect on the skin tissue, increasing the absorption of multi-component drugs by the cell body, and thereby performing the necessary specific immune regulation. Furthermore, it can also alleviate inflammation and promote tissue repair by activating Ca^2+^ signal transduction pathways (Fig. 6B). Compared with the group without drugs (ES) and the drug group, the Psoriasis Area and Severity Index (PASI) scores of the skin of rats with psoriasis in the mD-eMN group were lower (Fig. 6C). Furthermore, the epidermal thickness and Baker scores of rats with psoriatic skin in the mD-eMN group were relatively close to those in the control group (Fig. 6D, E). According to reports, this device can deliver drugs through the skin and, when combined with electrical stimulation, is highly effective for treating psoriasis through a synergistic approach.

These advanced therapeutic options collectively represent a significant advancement in the clinical management of psoriasis. The potential to revolutionize the personalized, patient-friendly, and efficacious treatment of this disease is demonstrated by continued efforts to integrate genetic, immunological, and intelligent drug delivery tools.

## Applications of MNs to Treat Atopic Dermatitis

Atopic dermatitis (AD) is another chronic inflammatory skin disease that affects 1%–3% of adults and 15%–20% of children worldwide. It leads to symptoms such as dry skin, abrasions, edema, bleeding, and severe erythema that can result in discomfort, loss of sleep, and reduced self-esteem [2, 73]. The application of MNs is a prospective method for the treatment of AD with the potential to enhance the safety and specificity of drug-based treatment strategies [73]. The applications of MNs in the treatment of AD are shown in Table 3. In this disease, MNs have been used as immune adjuvants capable of regulating hyperactive immune functions to alleviate the symptoms, while also exerting anti-inflammatory and antioxidant effects.

**Table 3:** Application of MNs for the treatment of atopic dermatitis

Types	Function	Action mechanisms	References
EGCG/AA-loaded MNs	EGCG/AA-loaded MNs maintained EGCG’s stability and efficiently delivered EGCG into the skin to ameliorate AD symptoms	EGCG/AA-loaded MNs dissolve and release EGCG and γ-PGA to exert antioxidant, anti-inflammatory and immunomodulatory effects	[76]
PLA-Pt-PPy MNs	PLA-Pt-PPy MNs enhanced the efficacy of transdermal drug delivery and offered a promising approach for transdermal drug delivery on demand	PLA-Pt-PPy MNs exert anti-inflammatory and immunomodulatory effects by releasing Dex drugs through electrical stimulation	[2]
TA–DMN	TA–DMN improved the poor solubility of TA	TA–DMN increases the drug loading of TA to exert anti-inflammatory and immunosuppressive effects	[73]
γ-PGA MNs	γ-PGA MNs can easily penetrate the epidermis and release γ-PGA into the dendritic cell-rich dermis to interact with dendritic cells for modulating immune responses	γ-PGA MNs dissolve and release γ-PGA to exert immunomodulatory effects	[77]
PLGA/HA MN	PLGA/HA MN improves both safety and efficacy in their daily use	PLGA/HA MN dissolves and releases CUR and GA to exert antioxidant and anti-inflammatory effects	[75]

EGCG/AA-loaded MNs, epigallocatechin gallate/L-ascorbic acid-loaded poly-γ-glutamate microneedles; EGCG, epigallocatechin-3-gallate; AA, L-ascorbic acid; AD, Atopic dermatitis; PLA-Pt-PPy MNs, polylactic acid-platinum-polypyrrole microneedles; PLA, polylactic acid; Dex, dexamethasone; TA, triamcinolone acetonide; TA–DMN, triamcinolone acetonide loaded in dissolving microneedles; γ-PGA MNs, γ-polyglutamic acid microneedles; γ-PGA, γ-polyglutamic acid; PLGA/HA MN, double-layered poly(lactic-co-glycolic acid)/sodium hyaluronate microneedle; PLGA, poly(lactic-co-glycolic acid); HA, hyaluronic acid; CUR, curcumin; GA, gallic acid

**Fig. 7:**
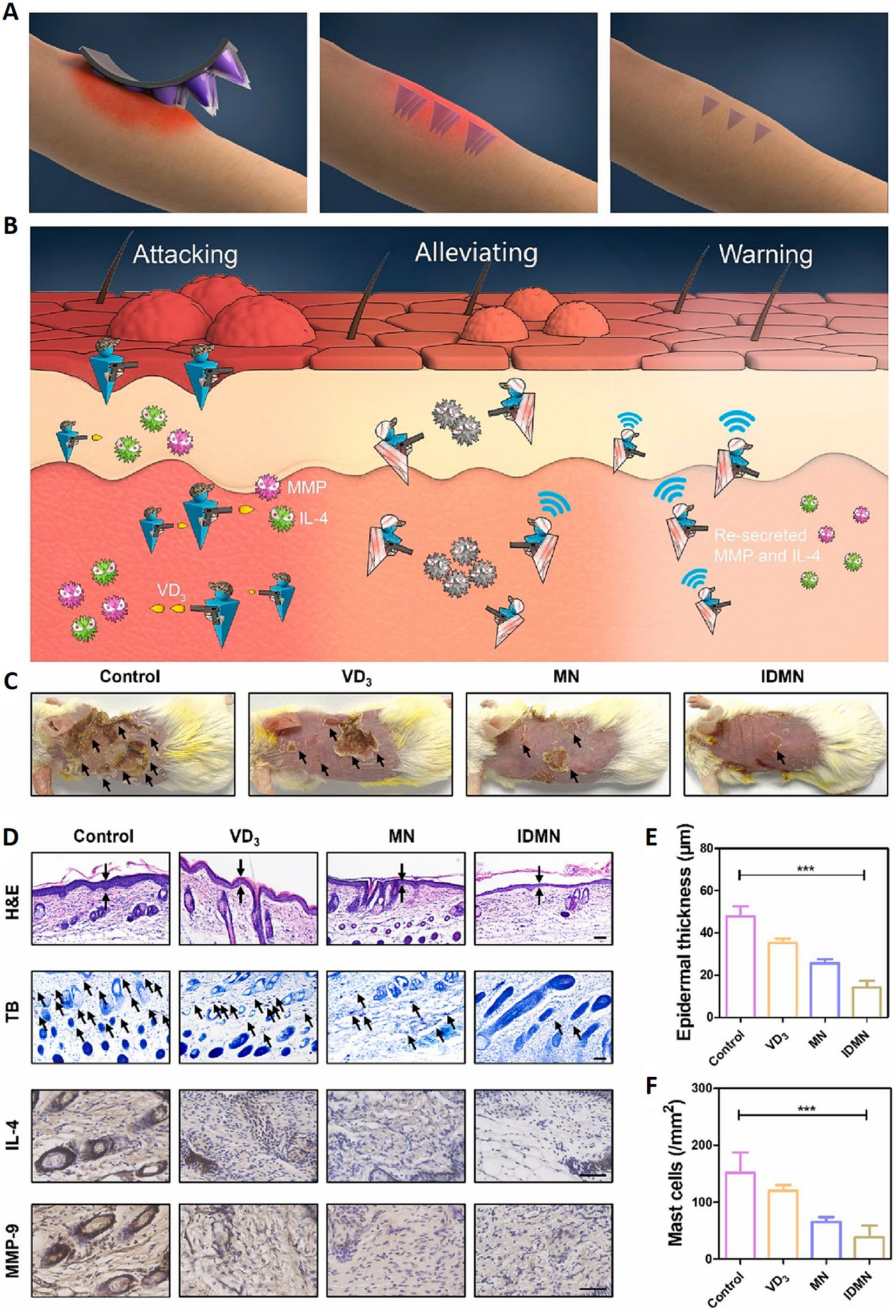
Application of an inflammation-responsive double-layer MNs platform to treat recurrent atopic dermatitis. **A** Inflammation-responsive double-layer MNs for the treatment of recurrent atopic dermatitis. **B** IDMN tips penetrating the skin are degraded through the stimulation of MMP, triggering the release of VD3 to resist the attack of IL-4 and MMP and alleviate the symptoms of AD. **C** Representative skin images of skin on the dorsal surfaces of mice in each group on day 12 after IDMN application. **D** H&E staining, toluidine blue staining, and immunohistochemical staining of IL-4/MMP-9 (scale bars are 50 μm). **E**, **F** Quantification of epidermal thickness and mast cell numbers in the different groups. *p < 0.05; **p < 0.01; and ***p < 0.001. Adapted from [74] with permission

Although the precise mechanistic basis for AD is only partially understood, numerous studies have shown a close correlation between this disease and an excessively active and aggressive immune response to specific environmental allergens or irritants. Researchers have developed an inflammation-responsive double-layer MNs (IDMNs) patch for the in situ delivery of Vitamin D3 (VD3) (Fig. 7A). VD3 has recently been shown to regulate immune activity through the induction of Treg responses, thereby maintaining the function of the epidermal barrier and associated cutaneous homeostasis, hence protecting against AD (Fig. 7B). Compared with the control group, the epidermal thickness of the IDMN group was significantly reduced from 47.8 to 14.1 μm, the number of mast cells was markedly reduced compared with the control group of 151.7 cells mm, and the expression of the inflammatory factor IL-4 and the MMP-9 content were significantly reduced (Fig. 7C–F). These significant improvements confirm the potential of IDMN patches in alleviating AD symptoms, inhibiting inflammatory responses, and preventing disease recurrence, providing a new and effective means for the treatment of AD [74].

T cell-mediated immune responses in the skin induce the secretion of inflammatory mediators, including MMPs and IL-4, during an exacerbation of AD symptoms, resulting in symptoms such as erythema and itching. MMP activity can degrade the tips of IDMNs after they have penetrated the epidermis, releasing significant quantities of VD3 to reduce further MMP- and IL-4-mediated symptoms. The resumption of MMP secretion will lead to the immediate degradation of any remaining GelMA tips within the epidermis when AD recurs in a treated patient. This process results in the release of VD3 as a therapeutic agent to protect against spontaneous AD recurrence. Few erythema, scaling, and lichenification symptoms were observed on the backs of mice in the IDMN group, suggesting that IDMNs had a more beneficial impact.

Recent reports have emphasized the importance of oxidative stress in the pathogenesis of AD. Phenolic compounds can restore redox balance, thereby reducing chronic oxidative stress and its associated inflammation by the suppression of associated proinflammatory pathways. A double-layered poly(lactic-co-glycolic acid) (PLGA)/sodium hyaluronate (HA) MNs patching has been developed to take advantage of this process. This patching can enable the sustained distribution of the natural polyphenols curcumin (CUR) and gallic acid (GA). The release of GA is triggered by the rapid dissolution of the HA layer within minutes following the insertion of the MNs into the skin, while the remaining PLGA tips can remain embedded within the dermis, sustaining the release of CUR for up to 2 months [75]. This was found to effectively ameliorate AD-like symptoms and combat 2, 4-dinitrochlorobenzene (DNCB)-induced oxidative stress in mice. This approach demonstrated significant potential in improving drug retention and achieving long-term durable treatment for AD.

There have been several other reports highlighting the benefits of intelligent MNs systems capable of mediating controlled drug release. Specifically, these systems are susceptible to various physiological changes associated with AD, including altered enzymatic activity or pH levels within the skin [76]. To more effectively treat the condition with fewer adverse effects, further refinements in the targeting of these MNs platforms are currently being explored for the treatment of acute and chronic AD through the controlled on-demand delivery of drugs via voltage modulation or other approaches.

## Applications of MNs to Treat Vitiligo

Vitiligo is a pigmentary skin disorder that affects 1% of the global population. It is characterized by milky white patches or macules that typically develop before the age of 20, with similar rates in both males and females [78]. However, there are currently no straightforward or effective treatment strategies for vitiligo, despite the detrimental impacts of the condition on the physical and mental health of affected individuals [79]. The most prominent models of vitiligo pathogenesis focus on melanocyte damage and the development of inappropriate autoimmune responses within skin lesions [80]. Despite intensive efforts to employ immune inhibitors, glucocorticoids, and narrow-band Ultraviolet B (UVB) phototherapy as a treatment for vitiligo, the cure rates remain low and treatment cycles are unsatisfactorily prolonged.

MNs may be employed as a monotherapeutic approach to vitiligo management or in conjunction with other topical agents, particularly for resistant regions or areas that are more challenging to treat, such as the extremities. The primary focus of current treatment initiatives is the integration of drugs and adjuvant therapies in clinical trials. For instance, oral Janus kinase (JAK) inhibitors, including tofacitinib, have shown potential when used in conjunction with UVB phototherapy, thereby facilitating the restoration of color in affected skin. The synthesis of melanin is further restricted in patients with vitiligo due to the low levels of alpha-melanocyte-stimulating hormone (α-MSH), which contributes to the depigmentation of the skin. In one report, researchers developed a separable hydrogel MNs capable of delivering α-MSH and tofacitinib as a treatment for vitiligo [79]. This hydrogel MNs was composed of dextran methacrylate (DexMA) and cyclodextrin-adamantane-based host–guest supramolecules (HGSM). α-MSH can enhance the therapeutic efficacy in patients with vitiligo by promoting the production of melanin. After two weeks of MNs treatment, the vitiligo scores of the control group and the tacrolimus ointment-treated group were still higher than 3 points, while the scores of the mice treated with the HGDexMA/α-MSH/tofacitinib MNs had even dropped to about 1 point. This phenomenon was more apparent during the follow-up treatment. The vitiligo score of the HGDexMA/α-MSH/tofacitinib MNs treatment group was reduced to 0 points MNs after 4 weeks of treatment, indicating that these are a tolerable and effective tool for treating vitiligo. Therefore, there are numerous possibilities for further innovative research focusing on the application of MNs-based platforms for more effective management of this disruptive disease. Additional advances in this therapeutic field are expected to pave the way for the more reliable treatment of vitiligo in future.

## Applications of MNs to Treat Hypertrophic Scarring

Hypertrophic scarring (HS) is a disorder associated with wound healing and is characterized by inappropriate fibroproliferation that imposes a substantial global healthcare burden [81, 82]. The development of HS can be attributed to the pathological repair of skin damage resulting from trauma, burns, or surgical incisions. These scars are often characterized by thick, red, hardened skin plaques with accompanying pruritis, pain, and impaired function that can impose cosmetic, psychological, and physical problems on affected individuals [82]. The three most prevalent pathological hallmarks of HS are excessive proliferation of hypertrophic scar fibroblasts (HSFbs), ECM dysregulation, and irregular collagen deposition. Therefore, these three factors are the primary targets for clinical treatments addressing this condition [83]. Conventional treatment strategies include surgical resection, drug-based therapy, and physical approaches such as radiotherapy, laser therapy, or silicone gel sheeting [84]. However, these approaches are limited by the need for an extended treatment interval, the potential for severe side effects, high rates of recurrence, and poor efficacy [85].

Improving multi-component drug delivery through the use of MNs is a promising strategy for the treatment of HS. The applications of MNs in treating HS are shown in Table 4. Researchers have explored the delivery of biological material-encapsulated hydrophobic drugs [86], the biphasic research of drugs through DMNs fabrication [84], and the revolutionary integration of TCM within MNs [87].

**Table 4:** Applications of MNs in hypertrophic scarring

Types	Function	Action mechanisms	References
HAase/ALA co-loaded MNs	HAase/ALA co-loaded MNs enhanced the efficiency of transdermal delivery of ALA, with increased cytotoxicity	HAase/ALA co-loaded MNs enhance the transdermal delivery of ALA, while the combination of HAase and Met can block autophagy and strengthen the anti-scar effect of PDT	[85]
TA-5-Fu-BMN	TA-5-Fu-BMN could coexist in the scar tissue for a sufficient period due to the well-designed biphasic release property	TA-5-Fu-BMN can downregulate the expression of Col I and TGF-β1 mRNA and proteins	[84]
BSP-MNs-QUE@HSF/CDF	BSP-MNs-QUE@HSF/CDF enhanced the therapeutic effect and provided a promising strategy for improving homogeneous targeted drug delivery of poorly water-soluble drugs and targeted therapies for skin diseases	BSP-MNs-QUE@HSF/CDF regulates the Wnt/β-catenin and JAK2/STAT3 pathways and reduces the expression of collagen I and III in HS	[87]

HAase/ALA co-loaded MNs, hyaluronidase /5-aminolevulinic acid co-loaded microneedles; HAase, hyaluronidase; ALA, 5-aminolevulinic acid; TA-5-Fu-BMN, triamcinolone acetonide-5-fluorouracil bilayer dissolving microneedle; TA, triamcinolone acetonide; 5-Fu, 5-fluorouracil; Col I, Collagen I; TGF-β1, transforming growth factor-β1; BSP-MNs-QUE@HSF/CDF, the hydrocortisone succinate film (QUE@HSF/CDF) coated with diphenyl carbonate cross-linked cyclodextrin metal–organic framework (CDF) containing quercetin (QUE) was dispersed in soluble microneedles prepared from Bletilla striata polysaccharide (BSP); BSP, Bletilla striata polysaccharide; QUE, quercetin; CDF, cyclodextrin metal organic framework; HSFs, hypertrophic scar fibroblasts; HS, hypertrophic scars

**Fig. 8:**
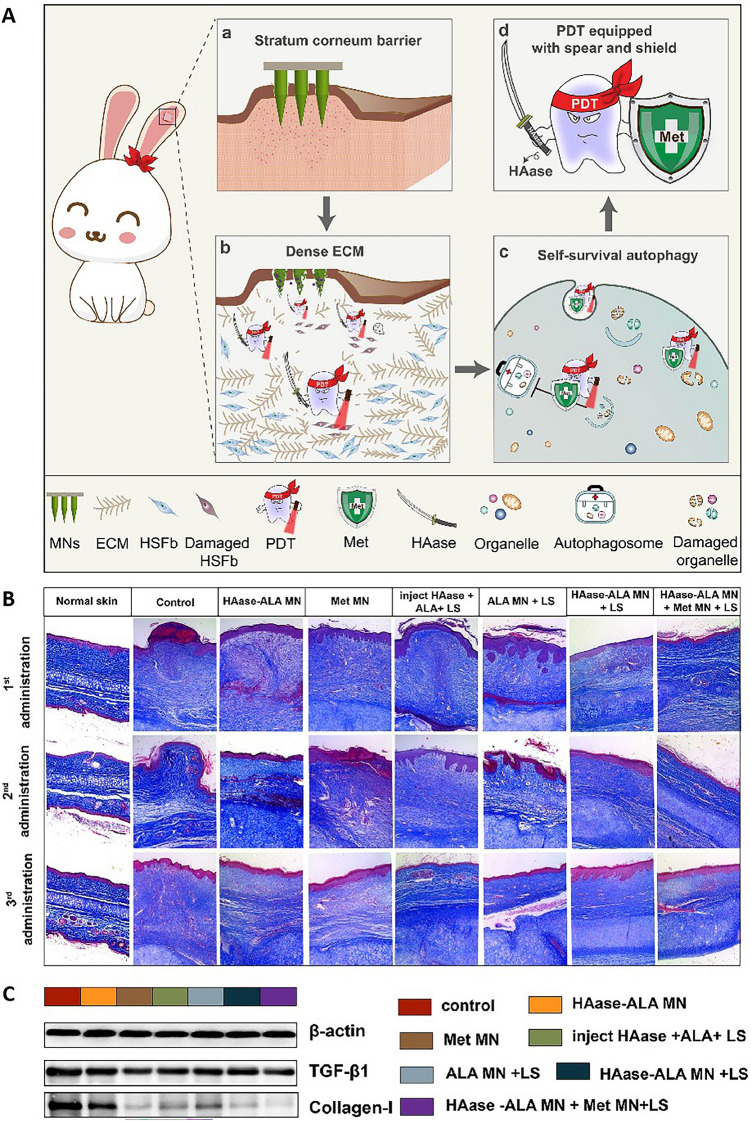
**A** Fully armed photodynamic therapy with spear and shear for topical deep hypertrophic scar treatment. **a** The MNs penetrate the stratum corneum (SC), thereby delivering drugs to the designated HS lesions. **b** HAase-based MNs were able to serve as a spear to facilitate the delivery of additional ALA within deep lesions by combatting ECM dysregulation, thereby improving photodynamic therapy (PDT) efficacy. **c** Met was employed as a shear to disrupt autophagy-mediated survival, further improving PDT efficacy. **d** The combined spear- and shear-based approach yielded a better-equipped PDT strategy equipped with the necessary tools to overcome the treatment resistance. **B** Masson’s trichrome-stained images of HS. **C** Western blots showing the expression of TGF-β1 and collagen I proteins in HS on completion of the experiments. Adapted from [85] with permission

MNs have also been assessed as potential tools for the combination treatment of scars in conjunction with other therapeutic modalities to achieve superior healing activity. For example, 5-aminolevulinic acid (ALA)-mediated photodynamic therapy (PDT) has been developed as a promising treatment for HS. In one study, researchers developed hyaluronidase (HAase)-based dissolving MNs as a "spear" that could deliver ALA deep within lesions, bypassing the SC and dense ECM found in HS lesions (Fig. 8Aa, Ab) [85]. HAase-ALA MN showed a cumulative drug penetration rate of nearly 100%, which was significantly better than 50% of ALA MN alone, indicating that the addition of HAase significantly improved the deep skin penetration of the drug. Metformin (Met) MNs can be used as a "shear" for the treatment of autophagy and respiration processes, thereby enhancing the efficacy of PDT (Fig. 8Ac, Ad). In addition, the combination of HAase-ALA MN and Met MN, supplemented by laser irradiation (LS), performed well in reducing the scar lift index (SEI), which decreased from 2.23 to 1.52, and further reduced the expression of TGF-β1 and collagen I, confirming the significant effect of the therapy in reducing scar height and improving the appearance of the skin (Fig. 8B, C). These findings confirm the potential of HAase and Met in enhancing the efficacy of PDT, providing a new strategy for the clinical treatment of HS. The integration of MNs into this therapeutic setting presents a unique opportunity to expand the range of treatment options for HS.

## Applications of MNs to Treat Melanoma

The current rate of skin cancer prevalence is increasing due to the accelerated aging of the global population. The primary cause of skin cancer-related mortality is melanoma, although it accounts for only 1% of all skin cancer cases [88]. Cutaneous melanoma remains the deadliest form of malignant skin cancer characterized by rapid local growth, high rates of metastatic progression, and a poor overall prognosis [89]. It is also the most aggressive type of skin cancer [88]. The efficacy of traditional treatments for melanoma including surgery, chemotherapy, radiotherapy, immunotherapy, and PDT is limited due to their associated severe side effects [90]. Both the progression and malignancy of melanoma are susceptible to pathophysiological factors, such as inflammation, and external environmental factors, such as radiation. The mutation of healthy cells can increase the risk of subsequent tumorigenesis, potentially increasing malignancy or the development of metastatic disease, although traditional chemotherapeutic and radiotherapeutic interventions are capable of destroying melanoma cells [89]. Therefore, it is imperative to develop a highly effective method for the elimination of melanoma cells and reducing the risk of tumor progression.

The potential for side effects can be reduced by the transdermal delivery of hydrophobic drugs by MNs, which demonstrate great promise as tools for improving therapeutic benefits. The applications of MNs in the treatment of melanoma are shown in Table 5. MNs can facilitate multi-component encapsulation, thereby allowing for synergistic efficacy [91, 92]. This is essential for achieving superior treatment outcomes by the efficient delivery of water-insoluble drugs through the skin. A hybrid peptide-saccharide material capable of CUR loading was developed through host–guest interactions by modifying GelMA side chains with amphiphilic β-cyclodextrin (β-CD) [93]. The structure was found to be extremely stable, enabling the controlled and sustained release of the loaded drug. Furthermore, biocompatibility was assessed by topical application to mouse skin. The GelMA-β-CD MNs penetrated the epidermal layer of the epidermis one hour after local implantation. Within 3 days, no inflammation or injury associated with the MN array was observed.

**Table 5:** Applications of MNs in melanoma

Types	Type of MNs	Type of nanocarriers	Strategy of treatments	Function	References
L-Ce6 MNs	DMNs	/	PDT	L-Ce6 MNs showed superior tumor-targeting efficacy and provided accurate and efficient delivery of L-Ce6 NAs to melanoma tumor lesions	[95]
5-Fu-ICG-MPEG-PCL@HA MN	DMNs	MPEG-PCL	PTT + Chemo	The MN enhanced the cure rate, controlled the release of 5-Fu to achieve a single-dose cure, and provided a novel idea and possibility for the treatment of skin cancer	[90]
FER@MN	DMNs	Self-assembled Nanofibers	Immunotherapy	FER@MN allowed controllable release and retention enhancement of the targeting peptide in the TME, thus efficiently inhibiting melanoma growth	[94]
GelMA-β-CD/CUR MN	Hydrogel MNs	GelMA-β-CD	Chemo	The therapeutic efficacy of the GelMA-β-CD/CUR MN was relatively higher due to its more localized and deeply penetrative nature	[93]
D/I@PATC MNs	DMNs	PATC	PTT + Chemo	D/I@PATC MNs not only enabled the visualization and verification of drug release from MNs but also facilitated the improvement of photothermal stability and reliability both in vitro and in vivo	[88]
PNA-DOX@ PDA-DOX@DMNs	DMNs	PNA + PDA	PTT + Chemo	The MNs induced the thermal ablation of tumor cells, resulting in the rapid release of drugs and providing a new synergetic chemo-photothermal strategy	[46]
PTX/IR-780 SLNs @DMNs	DMNs	Thermal-sensitive SLNs	PTT + Chemo	The MNs were released and uniformly distributed at the site, and achieved temporally controlled multiple dosages in a single administration	[97]
HRMAM	DMNs	PCL	Chemo	HRMAM achieved on-demand thermal-activated deep drug delivery, allowed for multi-round precise intervention without mutual interference, and concurrently endowed the HRMAM with enhanced geometrical adaptability at the irregular lesions	[96]

L-Ce6 MNs, Chlorin e6 Microneedles; NAs, nanoassemblies; 5-Fu, 5-fluorouracil; MPEG, monomethoxy-poly (ethylene glycol) polycaprolactone; ICG, indocyanine green; TME, tumor microenvironment; FE, PD-L1 targeting peptide; FER, FE self-assembled spherical micelles can efficiently encapsulate the immunologic adjuvants resiquimod (R848); β-CD, β-cyclodextrin; CUR, Curcumin; PATC, poly-AM-TPE-CAA; PNA–DOX@PDA–DOX, The nanoparticles have a doxorubicin (DOX)-loaded poly(*N*-isopropylacrylamide-*co*-acrylic acid) (PNA) nanogel core and a DOX-loaded polydopamine (PDA) outer layer; PTX/IR-780 SLNs @DMNs, Paclitaxel/IR-780 solid lipid nanoparticles in dissolving microneedles; DMNs, dissolving microneedles; SLNs, solid lipid nanoparticles; PTX, Paclitaxel; HRMAM, hydrothermally responsive multi-round acturable microneedle

**Fig. 9:**
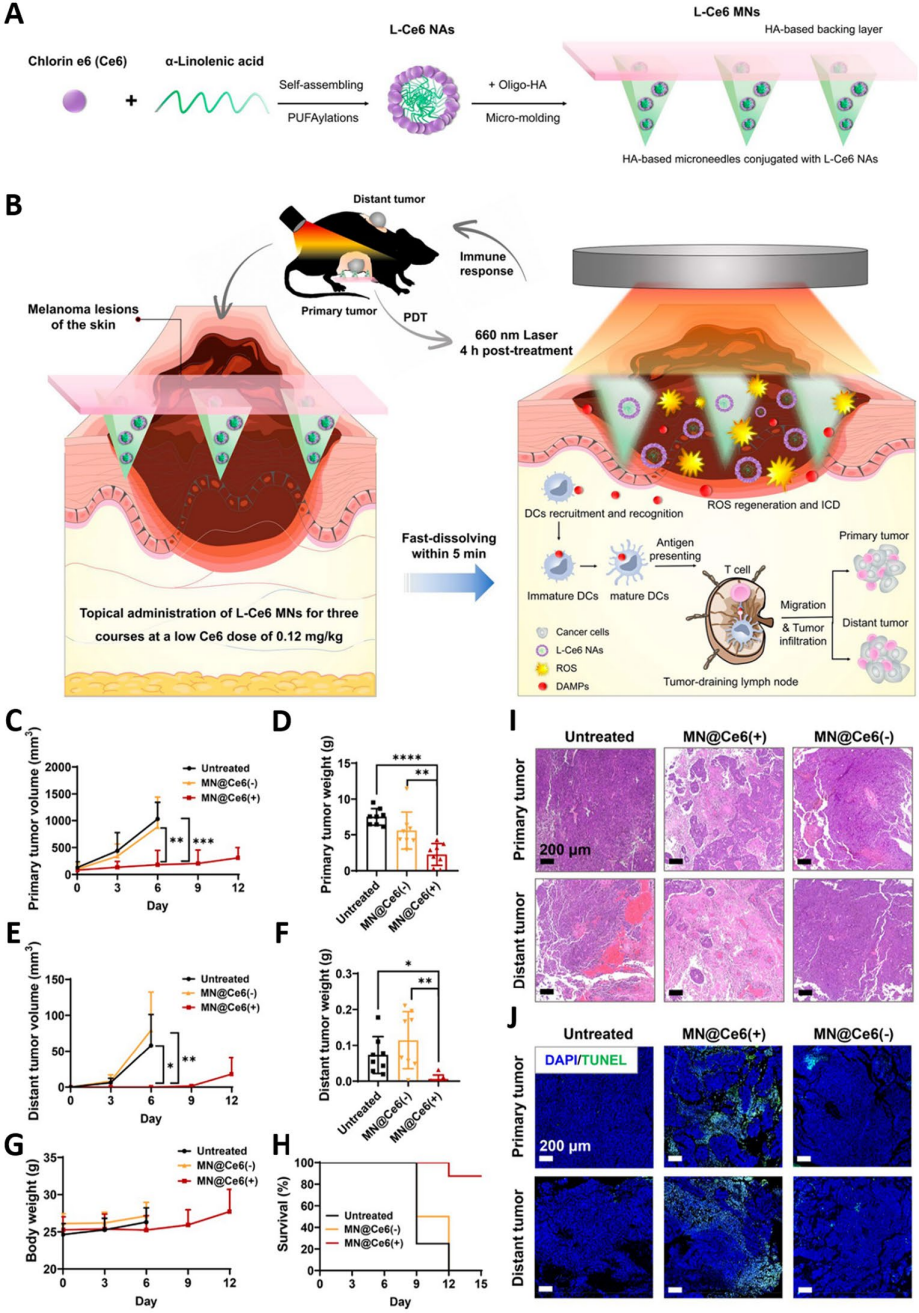
Schematic overview of the implementation of a rapid-dissolving MNs-based composite system employing low-dose photosensitizers for enhanced PDT-induced antitumor immunity. **A** Approach used for the L-Ce6 MNs fabrication. **B** L-Ce6 MNs were able to successfully deliver L-Ce6 Nas within target tumors in a bilateral model of melanoma, enabling ROS-mediated ablation of the primary tumor ablation upon laser irradiation at 660 nm. These L-Ce6 MNs were then able to trigger antitumor immunity via the initiation of immunogenic cell death (ICD) and associated damage- associated molecular patterns (DAMP) release, increasing the infiltration of bilateral tumors by T cells while inhibiting distant tumor growth. **C** Tumor growth curves and **D** tumor weights for the primary tumors in each treatment group. **E** Tumor growth curves and **F** tumor weights for the distant tumors in each treatment group. Monitoring of tumor growth for a treatment group was stopped when one mouse in the group died. Animals were euthanized when the size of tumors exceeded 2000 mm^3^. **G** Body weight changes of mice in each treatment group. **H** Kaplan–Meier survival curves for the treated and control mice. **I** H&E and **J** TUNEL staining analyses of both the primary and distant tumors of mice in each treatment group. Data are presented as mean ± SD (*n* = 8). *p < 0.05; **p < 0.01; ***p < 0.001 and ****p < 0.0001. Adapted from [95] with permission

MNs can also facilitate the delivery of drugs deep into tumor tissues to maintain redox homeostasis as an aspect of antitumor treatment, thereby inhibiting the progression of melanoma. Therefore, an increasing number of studies have attempted to exploit MNs to enhance the efficacy of immunotherapy or to improve other combination regimens for the treatment of melanoma. Current research endeavors were designed to encapsulate immune adjuvants in MNs to assist in the treatment of melanoma by preparing nanocarriers [89, 94] or nanovaccines [12]. Although immunotherapeutic approaches present a promising opportunity to eradicate melanoma, clinical response rates appear to be unsatisfactory. PDT has attracted significant interest as a treatment for melanoma due to its minimally invasive, convenient, and spatiotemporally controlled localized skin administration. Photosensitizer-containing nanosystems represent a hotspot in PDT treatment. Researchers have successfully fabricated dissolvable MNs L-Ce6 MNs of carrier-free nanoassemblies L-Ce6 NAs using a simple micromolding technique, which exhibited good mechanical strength and rapid drug release (Fig. 9A). L-Ce6 MNS PDT-based low-dose Ce6 (0.12 mg kg^−1^) was found to effectively ablate the primary lesion in situ through the production of reactive oxygen species (ROS), as well as significantly promoting dendritic cell maturation and T cell-mediated immune responses, improving the uptake efficiency of B16F10 melanoma cells, and inducing concentration-dependent cytotoxicity and immunogenic cell death (ICD) (Fig. 9B). Compared to the untreated group and the MN@Ce6 (−) group treated with microneedling alone, the MN@Ce6 (+) group receiving L-Ce6 MNs and light irradiation demonstrated significant antitumor effects, including an inhibition of primary tumor growth and prolongation of survival. Treatment with MN@Ce6 (+) not only effectively inhibited the growth of the primary tumor, but also activated the systemic antitumor immune response, significantly inhibiting the growth of distant tumors (Fig. 9C–J) [95]. Therefore, immune adjuvant integration into MNs platforms represents a highly advantageous immunotherapeutic strategy. This innovative method provides a strong basis for clinical translation, providing an essential development in the more precise and effective treatment of melanoma.

**Fig. 10:**
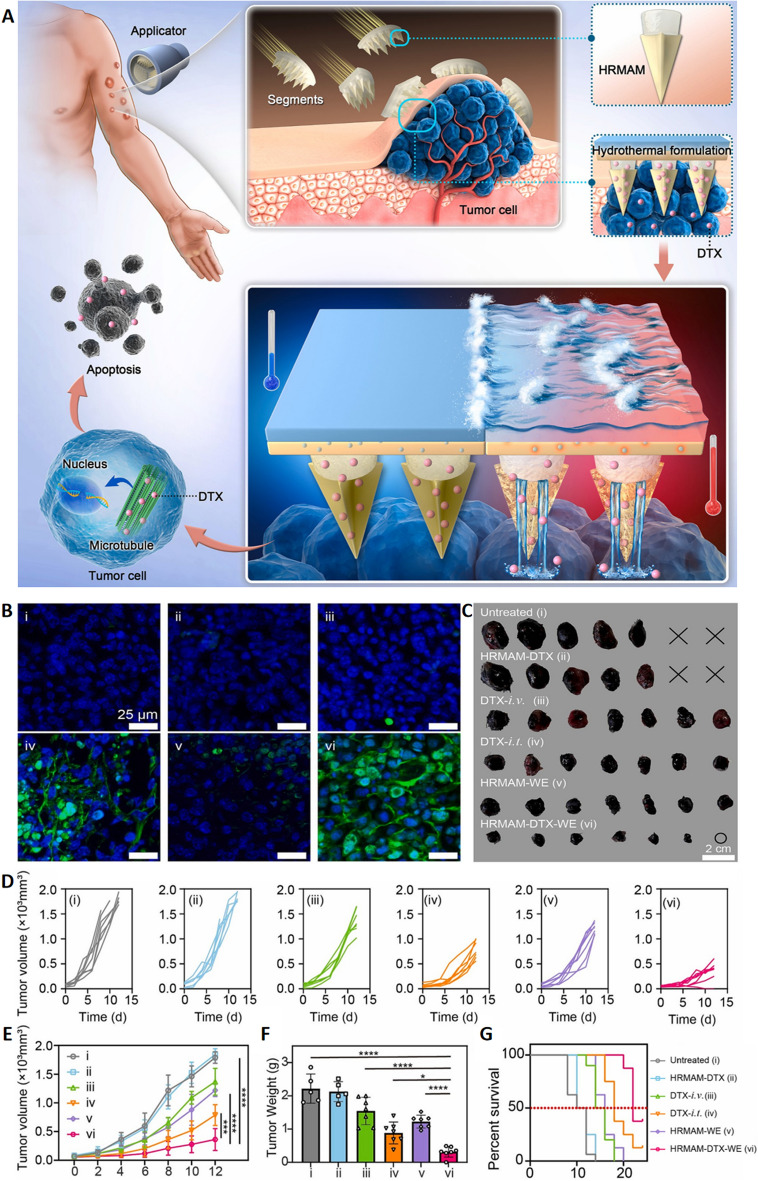
**A** HRMAM design and implementation. **B** Analysis of cell apoptosis in the tumor tissues after different treatments. **C** Tumors were harvested from each group. **D** Individual tumor growth curves of B16F10 tumors after various treatments (*n* = 7). **E** Average tumor growth curves (*n* = 7). ***p < 0.001 and ****p < 0.0001. **F** Tumor weights in each group on day 12 (*n* = 7). *p < 0.05; **p < 0.01; ***p < 0.001; and ****p < 0.0001. **G** Survival rates of the mice in each group. *p < 0.05; **p < 0.01; ***p < 0.001; and ****p < 0.0001. Adapted from [96] with permission

Locoregional treatment approaches have been recognized as promising approaches to managing subcutaneous tumors. The integration of MNs into combined therapeutic regimens represents an attractive and advantageous approach for melanoma management [62, 92]. It has been consistently demonstrated that combination therapies are more effective in the treatment of melanoma. Photothermal therapy (PTT) has been demonstrated to be a successful approach to tumor ablation. The development of drug and photothermal agent-loaded MNs is an essential step in the more effective treatment of melanoma patients [90]. MNs can be administered with superior precision, allowing for the precise targeting of tumors in a minimally invasive and largely painless manner. Researchers developed a three-segment hydrothermally responsive multi-round acturable MNs (HRMAM) system that could be loaded into an appropriately designed applicator for fixation to target tumors. The HRMAM system was capable of generating heat when triggered by water, inducing the glass transition of Poly ε–caprolactone (PCL), and achieving on-demand drug release. The grooved structure at the tip of the MNs facilitates deep penetration of drugs into tumor tissue, enhancing delivery efficiency (Fig. 10A). The tumor sizes and weights of the treated mice validated the therapeutic effect of the HRMAM-DTX-WE group. The results of the TUNEL staining showed that the apoptosis level was the highest in the tumor tissues of HRMAM-DTX-WE-treated mice (Fig. 10B, C). The tumor growth inhibition rates of the intravenous DTX (DTX-i.v.) and HRMAM-WE groups were 23.44% and 31.52%, respectively, both superior to those of the untreated group but lower than the intratumoral DTX (DTX-i.t.) group at 53.20%. This indicates that single heat-induced cell killing or limited drug accumulation alone is insufficient to effectively combat tumors. The HRMAM-DTX-WE group showed the best therapeutic effect, attributed to the heat generated by on-demand multiple rounds of water heating, together with deep penetration and drug delivery based on MNs (Fig. 10D). The HRMAM-DTX-WE group showed a tumor growth inhibition rate (TGI) of 75.11%, significantly higher than intravenous DTX (DTX-i.v.) and intratumoral DTX (DTX-i.t.) injections(Fig. 10E–G) [96]. The synergistic combination of chemotherapy and PTT thus has the potential to be a comprehensive and effective strategy for treating melanoma. This approach ensures the multidirectional attack of melanoma cells, which is mediated by the enhanced sensitivity of tumor cells to chemotherapeutic treatment and the damage-inducing heat generated by photothermal agents. Therefore, the risk of tumor growth and dissemination is reduced.

**Fig. 11:**
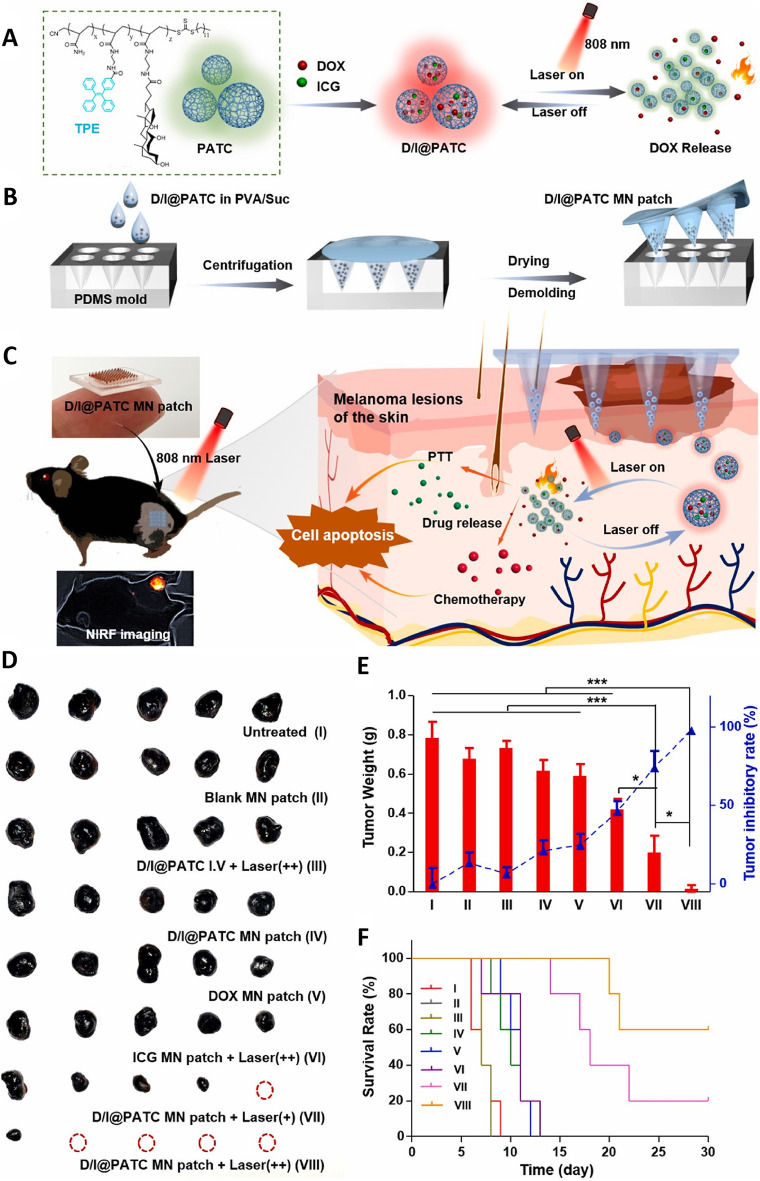
**A**–**C** Schematic overview of the use of D/I@PATC MNs patches for the laser-triggered synergistic chemo-photothermal treatment of melanoma. **D**–**F** Antitumor efficacy evaluation of the D/I@PATC MN patches in B16–F10 melanoma modeling mice (*n* = 5). *p < 0.05 and ***p < 0.001. Adapted from [88] with permission

The emergence of intelligent MNs has been a significant advancement in the treatment of melanoma, potentially leading to more effective results. By leveraging phase-change materials or specialized materials with unique optical properties to achieve drug loading, these MNs are responsive to external stimuli, thereby enabling the controlled delivery of drugs, which is crucial for long-term therapeutic efficacy. There has been substantial interest in treating melanoma through a nanomedicine-based PDT approach. Based on this, researchers have developed a self-monitoring MNs-based drug delivery platform integrating aggregation-induced emission (AIE)-active poly-AM-TPE-CAA (PATC) microparticles into MNs patches to treat melanoma via a light-controlled pulsatile chemo-photothermal synergistic approach. The utilized PATC microparticles (D/I@PATC) were developed by encapsulating indocyanine green (ICG), a photothermal agent, with doxorubicin (DOX), a chemotherapeutic drug (Fig. 11A, B). Under 808 nm laser irradiation, D/I@PATC particles achieve controlled release of DOX through the switching cycle of light, which can be quickly and efficiently delivered to tumor sites in vivo, and show stronger photothermal stability and longer retention time than ICG MN patches (Fig. 11C). In terms of the anti-melanoma effect in vivo, the D/I@PATC MN patch combined with two laser irradiation significantly inhibited tumor growth and improved the survival rate of mice without causing significant systemic toxicity. Compared with other control groups, the D/I@PATC MN patch + Laser (++) showed superiority in antitumor effect, photothermal stability and drug release control (Fig. 11D–F) [88]. In a separate study, researchers developed a spatiotemporally controlled pulsatile release system in which thermally-sensitive solid lipid nanoparticles (SLNs) were used to encapsulate paclitaxel (PTX) together with the photothermal agent IR-780. The SLNs were then concentrated in DMNs tips (PTX/IR-780 SLNs@DMNs) to suppress tumor growth through the synergistic chemotherapeutic and photothermal effects of the DMNs and SLNs [97]. This system facilitated the sustained release of drugs over extended periods. These PTX/IR-780 SLNs@DMNs used innovative phase-change materials, which enabled them to modulate their state in response to changes in temperature. The resultant intelligent MNs were suitable for direct cutaneous application, eliminating the requirement for systemic drug delivery and thereby reducing the possibility of treatment-associated adverse effects.

## Applications of MNs to Treat Acne

Acne is a prevalent chronic inflammatory skin condition that impacts more than 80% of the worldwide population, rendering it one of the most common skin disorders. This condition is initiated by multiple factors, including genetics, hormones, infections, and environmental elements. The primary mechanism responsible for the development of acne is the excessive production of sebum by the sebaceous glands, which leads to the obstruction of the sebaceous gland unit, which includes follicles, hair shafts, and sebaceous glands [98]. Prolonged facial acne scarring and hyperpigmentation may be the consequence of severe inflammation in certain cases, which could have significant effects on the mental health and physical appearance of the affected person [99]. Oral antibiotics are the primary treatment for acne. However, their widespread use poses concerns regarding the development of drug resistance. Furthermore, topical antimicrobial products frequently demonstrate inadequate bacterial eradication because of their inability to penetrate the skin effectively, resulting in limited efficacy. MNs are a promising approach for improving the delivery and efficacy of antibiotics in the treatment of acne by enhancing their skin penetration properties.

Clinical trials are the primary focus of research on the treatment of acne by MNs. These trials have investigated the use of MNs as a drug delivery mechanism to transport anti-acne substances through the SC and thus enhance their effectiveness. Furthermore, there are other studies various studies that used MNs loaded with functional substances to assist external forces to treat acne [36] or used MNs to target the dermis and reduce drug depletion in non-active areas [100].

Infection by the anaerobic bacterium *Propionibacterium acnes* is the main cause of acne. A study reported the synthesis and loading of a composite structure of zinc oxide (ZnTCPP@ZnO) and zinc porphyrin-based MOF into sodium hyaluronate-synthesized MNs to treat acne infection. The results also showed that activated oxygen-mediated eradication of P. acnes had an antibacterial efficiency of 99.73% under 15 min of ultrasound irradiation. This resulted in a reduction in acne-related variables and promoted the growth of fibroblasts, ultimately facilitating skin repair [36].

MNs are a promising and well-tolerated treatment option that appears to decrease acne scarring. The application of MNs reduces the frequency of administration and prevents the reduction of bacterial sensitivity to drugs, overcomes the epidermal barrier, and improves drug permeability. Furthermore, the combination of MNs with additional antibacterial drugs is expected to enhance acne treatment by integrating adjuvant therapies and expanding the application of MNs.

## Applications of MNs to Treat Skin Infections

Skin infections caused by pathogens, including bacteria, fungi, and viruses, are difficult to eliminate through standard topical administration due to low bioavailability at the infection site, lack of sustained therapeutic effect, and the development of drug resistance, resulting in significant economic and medical burdens. The use of MNs as a precise drug delivery system for fungal infections has attracted significant attention in recent years [101, 102]. The applications of MNs in skin infections are shown in Table 6.

**Table 6:** Applications of MNs in fungal and bacterial skin infections

Diseases	Types	Types of MNs	Loaded agents	Function	References
Skin fungal infections	CuS/PAF-26 MN	DMNs	Copper sulfide nanoenzyme and antimicrobial peptide	CuS NE catalyzed hydrogen peroxide to produce ROS, and PAF-26 directly destroys the cell membrane of fungi. The combination of ROS toxicity produced by CuS NE and the destruction of fungal membranes by PAF-26 show strong antifungal activities without causing drug resistance	[103]
	AmB DMP	DMNs	Amphotericin B	The work introduced a novel and straightforward DMP system that can be easily fabricated and is capable of delivering hydrophobic drugs intradermally with prolonged and localized release profiles, as well as less systemic exposure. This system has the potential to be extensively used for effective and toxic hydrophobic therapies	[104]
	PILMN-NO	Coated MNs	NO	Upon the insertion of the PILMN-NO into the skin, the contact fungicidal activities induced by electrostatic and hydrophobic effects of the poly(ionic liquid) and the released NO sterilization resulting from the peroxidation and nitrification effect of NO achieved enhanced antifungal efficacy against fungi both in vitro and in vivo. Simultaneously, the PILMN-NO showed biofilm ablation ability and efficiently eliminated mature biofilms, effectively sterilized fungi while suppressing the inflammatory reaction, facilitated collagen deposition and angiogenesis, and promoted wound healing	[105]
Skin bacterial infections	DOX-EPL MNs	DMNs	ε-poly-L-lysine and doxycycline	The formation of physically cross-linked networks of EPL provided MNs with improved formability, good mechanical properties, and amorphous nanoparticles of encapsulated DOX. Furthermore, it provided potent, long-term effects by synergistically enhancing antibiotic activities and prolonging drug retention in infected lesions	[106]
	Hybrid MN arrays	Hybrid MNs	Vancomycin	The hybrid, two-layered MN arrays consisted of an outer water-soluble layer loaded with VAN and an inner water-insoluble near-IR photothermal core, showed the inhibition of MRSA growth due to the synergistic interaction of heat with VAN-reducing the bacterial survival by up to 80%	[107]
	PEGDA@film–NS MNs	Coated MNs	Nanosilver	The PEGDA@film–NS MNs showed remarkable mechanical strength for effective skin insertion, and the loaded cargo can be rapidly released upon dissolution of the film under physiological conditions	[102]
	MgB_2_ MN	Integrated MNs	MgB_2_	MgB_2_ MN regulated the acidic microenvironment in bacteria-infected both subcutaneous abscesses and wounds. It also eradicated the bacteria and eliminated their remains to prevent local inflammation during wound healing	[108]
	PVAG MNs	DMNs	Bacteriophages	This phage delivery strategy presents further possibilities for the future development of phages with poor anti-biofilm activity to address local biofilm-associated lesions, particularly those with antibiotic-resistant pathogens	[109]
	NPs/IL dispersions of DMNs	DMNs	Zinc oxide nanoparticles	The DMN showed synergistic antibacterial activity	[110]
	LipoRIF-DMNs	DMNs	Rifampicin-loaded liposomes	LipoRIF-DMNs were able to resolve the issue of poor drug solubility and significantly improve skin deposition, but they also promoted undesired RIF transdermal absorption	[111]
	PET-PTFE-Based TENG MNs	DMNs	Antibiotics	The PET-PTFE-based TENG facilitated the real-time monitoring of wound progress, provided the necessary depth of skin penetration, and achieved a prolonged release of the drugs	[112]

CuS/PAF-26 MN, copper sulfide/antimicrobial peptide microneedle; CuS, copper sulfide; PAF-26, antimicrobial peptide; CuS NE, copper sulfide nanoenzyme; ROS, reactive oxygen species; AmB, amphotericin B; DMP, dissolving microneedle patches; PILMN-NO, nitric oxide (NO)-releasing poly(ionic liquid)-based microneedle; DOX-EPL MNs, doxycycline-ε-poly-l-lysine microneedles; DOX, doxycycline; EPL, ε-poly-l-lysine; VAN, vancomycin; MRSA, methicillin-resistant Staphylococcus aureus; PEGDA@film–NS MNs, film-coated poly(ethylene glycol) diacrylate-nanosilver microneedles; PEGDA, poly(ethylene glycol) diacrylate; NS, nanosilver; NPs/IL dispersions of DMNs, nanoparticles/ionic liquid dispersions of dissolving microneedles; NPs, nanoparticles; DMNs, dissolving microneedles; LipoRIF-DMNs, rifampicin-loaded liposomes-dissolving microneedles; LipoRIF, rifampicin-loaded liposomes; PET-PTFE-based TENG MNs, polytetrafluoroethylene-polymethyl-based triboelectric nanogenerator microneedles; TENG, triboelectric nanogenerator; PTFE-PET, polytetrafluoroethylene-polymethyl

In particular, copper sulfide nanoenzyme (CuSNE) demonstrated superior oxidase-like and peroxidase-like activities, which can be used to accomplish effective antimicrobial activity and prevent drug resistance by generating ROS. Interestingly, an antimicrobial peptide (PAF-26) was found to have good antifungal and cell-penetrating activities and does not easily lead to drug resistance. More importantly, the ability of PAF-26 to penetrate the cell envelope can facilitate CuS NE-generated ROS to enter fungi and overcome the defects of ROS. The study designed a HA-based biodegradable MN (CuS/PAF-26 MN) loaded with both CuS NE and PAF-26 for the treatment of deep cutaneous fungal infections without causing drug resistance [103]. The therapeutic efficacy of the CuS/PAF-26 MN was demonstrated by the fact that the amount of fungal residue was nearly identical to that of uninfected skin following treatment, whereas infection was still evident following treatment with other groups. Furthermore, the skin thickness was greater than that of normal skin following treatment with the CuS/PAF-26 MN, further demonstrating the superiority of this formulation.

The use of MNs for treating fungal or bacterial infections of the skin has improved the durability of commonly used drugs, addressed the challenges of poor solubility and permeability of insoluble drugs, and implemented the use of nanomaterials for the treatment of skin fungi or bacteria. This has expanded the concept of disease treatment and enhanced the systematic antibacterial performance of drugs.

## Clinical Trials of MNs

In recent years, there have been significant developments in the clinical incorporation of MNs into dermatological practice. In the context of skin disease treatment, MNs have been employed predominantly in conjunction with PDT or a variety of drugs to assist in the management of conditions such as psoriasis, acne, and melanoma (Tables 7, 8). Furthermore, MNs have the potential to induce a wound healing response in the skin through the formation of controlled microinjuries. This response may mobilize and activate melanocyte stem cells and precursors within the epidermal layer and HFs, thereby promoting the repigmentation of vitiliginous skin [113]. Indeed, numerous studies have demonstrated the beneficial effects of MNs when used alone or in conjunction with other treatments for vitiligo [113–115]. Tacrolimus is a topical immunomodulatory calcineurin inhibitor that has been employed to treat vitiligo. Studies have shown that the combination of tacrolimus with MNs therapy for vitiligo showed more rapid efficacy and more significant therapeutic effects than tacrolimus alone. Specifically, the pigment repigmentation rate of 50% of patients treated with the combination therapy was 75%, while that of patients treated with tacrolimus alone was only 29.2%. Moreover, the expression of c-kit in patients treated with the combination therapy was significantly higher than that in patients treated with tacrolimus alone. This indicates the superiority of the combination of tacrolimus with MNs therapy for vitiligo [116]. Therefore, MNs are increasingly being used in clinical practice due to their potential to assist in the treatment of diseases with drugs. Although microneedling has shown some efficacy in the treatment of vitiligo, there are still studies that suggest that microneedling does not appear to bring additional therapeutic value to NB-UVB phototherapy in the treatment of stable acral vitiligo, and both methods have a risk of folliculitis [117]. Therefore, future research directions may include an exploration of the optimal parameters of microneedling therapy (e.g., depth and frequency of microneedling), exploring its potential to promote pigment recovery by generating mechanotransduction signals through mechanical stimulation of microneedling, and the combination application of microneedling with other treatments (e.g., drug therapy, cell therapy, phototherapy, etc.) to improve efficacy and reduce side effects.

**Table 7:** Recent clinical trials focused on the treatment of skin diseases

Diseases	Types	Advantages	References
AGA	MNDS	MNDS provided superior overall changes in hair regrowth relative to saline solution	[120]
	PRP combined with dermapen MNs	The application of the combination therapy in AGA patients was superior to a point-by-point injection technique	[121]
	Topical minoxidil and nano-MNs-assisted FGF	Combination therapy was found to be preferable to monotherapy with respect to hair regrowth, and considered as a safe and reliable treatment for male AGA	[122]
AA	PRP combined with MNs	FCL and MNs could facilitate topical transdermal PRP delivery while reducing the pain associated with intradermal injection and preserving PRP efficacy	[123]
	MNs combined with topical vitamin D3 or bimatoprost	The combination therapy improved the absorption of both drugs and ensured their more uniform distribution, thereby having a more substantial effect on AA	[124]
	Self-DMNs patches embedded with corticosteroids	MNs demonstrated superior efficacy as a topical therapy	[125]
Psoriasis	An HA-based MNs patch	The application of an MNs patch was found to be significantly associated with improvements in psoriatic resolution	[126]
Vitiligo	MNs combined with pimecrolimus, 5-Fu, and TCA	Combination treatment was capable of enhancing therapeutic responses and decreasing the possibility of recurrence	[127]
	MNs injection combined with blood transfusion	Combination therapy demonstrated a significant improvement in patient blood indices and skin plaques, with a high degree of clinical acceptance	[128]
	MNs combined with tacrolimus versus calcipotriol plus betamethasone	MNs had an additive effect, enhancing the rate and the degree of pigmentation	[129]
AD	A BHMNs patch	The BHMNs patch was associated with an increase in topical drug absorption through the generation of MNs-based micropores penetrating the epidermis	[130]
Acne scars	MNs (Dermapen) combined with Jessner’s solution peeling	The combination technique was associated with the greatest clinical improvement and the fewest sessions, followed by MNs treatment alone. Jessner's solution peeling was the least effective	[131]
	MNs and subcision with PMMA-collagen gel injections	MNs were able to significantly improve the SGAIS and PGAIS scales	[132]
	MNs combined with 15% TCA peel versus 25% pyruvic acid peel	MNs resulted in the formation of microscale punctures in the dermis generated with a drum-shaped device with fine protruding needles, leading to the release of a range of growth factors that were able to assist the filling of atrophic scars via neovascularization and neocollagenesis	[133]
	PDLA using MFRF	MNs were associated with reduced pain and nerve injury as compared to intravascular injection	[134]
	MNs combined with PRP	A substantial increase in the production of types I, III, and VII collagen, as well as a decrease in the total amount of elastin, was observed at the end of the treatment	[135]

AGA, androgenetic alopecia; MNs, microneedles; MNsDS, MNs plus topical dutasteride solution; PRP, platelet-rich plasma; FGF, fibroblast growth factor; FCL, fractional CO_2_ laser; AA, alopecia areata; HA-based MNs, hyaluronic acid-based microneedles; 5-Fu, 5-fluorouracil; TCA, trichloroacetic acid; BHMNs, biodegradable hyaluronic acid MNs; AD, atopic dermatitis; PMMA, polymethylmethacrylate; SGAIS, subject-global aesthetics improvement scale; PGAIS, physician-global aesthetics improvement scale; PDLA, poly-D, L-lactic acid; MFRF, MNs fractional radiofrequency

**Table 8:** Recent clinical trials for skin disease treatment

Diseases	Study title	Medication	ClinicalTrials.gov Identifier	Status	Study date
Androgenetic Alopecia	Evaluating the Efficacy of Microneedling in the Treatment of Androgenetic Alopecia	Topical 5% Minoxidil (Microneedling)	NCT02154503	Phase 1	June 2014–March 2017
	Comparison of Microneedling vs. Autologous Concentrated Growth Factor for the Treatment of Female Androgenetic Alopecia	Procedure: MN Procedure: Autologous Concentrated Growth Factor Drug: 5% minoxidil	NCT06218394	Not applicable	December 2023–December 2024
Psoriasis	Analysis of Non-Invasively Collected MN Device Samples From Mild Plaque Psoriasis for Use in Transcriptomics Profiling	MN Device	NCT03795402	Completed	March 2019–December 2019
	MN Patch for Psoriatic Plaques	MN**-**HA patch	NCT02955576	Not applicable	September 2016–November 2017
Hypertrophic Scars	Fractional Microneedling RF vs Intralesional Steroid With & Without Microneedling in Hypertrophic Scars	Device: Fractional Microneedling Radiofrequency Procedure: Intralesional Steroid Injection with and without Microneedling	NCT04389619	Not applicable	May 2020–December 2020
	Comparison of Cosmetic and Functional Outcome of Silicone Sheeting and Micro-needling on Hypertrophic Scars	Procedure: Microneedling Procedure: Percutaneous Collagen Induction	NCT05108272	Not applicable	July 2021–March 2022
Acne	Clinical Trial of MN Radiofrequency Combined With Oral Isotretinoin in Moderate to Severe Acne	Drug: Oral isotretinoin Other: Oral isotretinoin combined with MN radiofrequency therapy	NCT06378983	Not applicable	September 2022–June 2024
	Trial of Single MN Radiofrequency for Moderate-to-Severe Acne Vulgaris	Other: Single MN Radiofrequency therapy Other: Photodynamic therapy	NCT04213638	Not applicable	November 2019–November 2020
	Comparison of 1,550-nm Laser and Fractional Radiofrequency MN for the Treatment of Acne Scars in Ethnic Skin	Device: Fraxel Restore Device: Fractora	NCT03380845	Not applicable	March 2018–October 2018
	Comparison of Treatments for Atrophic Acne Scars	Procedure: Laser Procedure: Microneedling	NCT02025088	Not applicable	December 2013–November 2014
	A Proof of Concept Study to Evaluate the Efficacy and Tolerability of Microneedling With SkinPen in Female and Male Subjects With Facial Acne Vulgaris, Ages 18 Through 45	Device: Skinpen Precision System	NCT05071274	Not applicable	March 2021–March 2022
	Comparison of Efficacy Between Fractional Microneedling Radiofrequency and Bipolar Radiofrequency for Acne Scar	Device: microneedling radiofrequency device Device: bipolar radiofrequency	NCT02207738	Not applicable	January 2012–May 2013

MN, microneedle; MN-HA, microneedle-hyaluronic acid; RF, radiofrequency

In summary, while there have been considerable advances in the potential use of MNs for the treatment of skin diseases and some instances of their clinical uptake, the limited variety of drugs suitable for MNs-based delivery remains a significant challenge [118]. MNs are not well-suited for the delivery of various pharmacological compounds, particularly those that have a high molecular weights or require specialized stability [44]. The development of biocompatible materials with specialized properties that can be used to fabricate more effective MNs is also a subject of current research. Although these materials are designed to be safe and not react with human tissues, it will be necessary to conduct comprehensive clinical trials to completely verify their use [119]. Furthermore, there is an urgent requirement for further research that emphasizes optimizing the design of MNs to achieve superior therapeutic efficacy and more efficient drug delivery, all while maintaining patient comfort, enhancing drug delivery efficiency, patient comfort, and overall treatment efficacy.

## Conclusions and Future Perspectives

The potential for normal tissue toxicity can be reduced by evading first-pass effects, and local drug utilization can be improved by using MNs to assist in topical drug delivery to target lesions via the skin [136]. The size and geometry of MNs can be modified based on the disease condition, and a simpler strategy is being pursued to prepare MNs with unique structures and reduced preparation costs. Furthermore, MNs therapy has not been associated with any inflammation in the treatment of skin diseases, as indicated by the most recent research. MNs are well-positioned as promising devices that can enhance patient quality of life by exploiting the advantages associated with a variety of biomedical, nanomaterial, nanomedical, and photonic technologies. This is due to the current advancement of novel intelligent treatment platforms and approaches [8]. MNs are anticipated to become a significant hotspot in the evolving nanotechnology and therapeutic landscapes due to their adaptability, painlessness, minimal invasiveness, accuracy, precision, and portability.

Globally, skin diseases continue to pose a significant burden on numerous individuals and society as a whole, underscoring the necessity of developing more effective treatments with superior therapeutic efficacy [44]. MNs-based treatment platforms have great potential for personalized treatment, allowing for the greater customization of the types of biomaterials, bioactive compounds, and drugs used to meet the clinical requirements of particular patients [137]. Furthermore, it is feasible to develop materials that are responsive to specific stimuli to facilitate the on-demand release of drugs, thereby enabling disease treatment. Other therapeutic strategies, such as CM and ICB, have the potential to be used in conjunction to achieve synergistic efficacy or to extend the associated therapeutic window. Significant progress has been made in the exploration of MNs as a clinical tool for treating various skin diseases. In androgenetic alopecia, they can promote the absorption of hair growth drugs and facilitate hair regeneration. In psoriasis, they can enhance drug transdermal delivery and alleviate symptoms. In atopic dermatitis, they can help anti-inflammatory drugs penetrate the barrier to reduce inflammation and itching. For scars, they can stimulate repair and improve appearance and function. In vitiligo, they can promote the effect of repigmentation drugs to facilitate skin repigmentation. In melanoma treatment, they can assist drug percutaneous absorption to improve therapeutic efficacy. In acne treatment, they can reduce sebum secretion, promote drug penetration to suppress inflammation and reduce scars. In skin infections, they can enhance the effects of anti-infective drugs and accelerate healing.

Although the clinical applications of MNs have many advantages, they also face some challenges. Material safety is the primary consideration. Although most existing MNs materials are biocompatible, long-term use or in specific populations (such as those with allergic constitutions) may still cause adverse reactions, such as local inflammation, allergic reactions, and granulomas caused by material residues [138]. The mechanical performance of MNs is poor. They are easy to break or bend during the insertion process, thus failing to penetrate the SC [139]. Metal MNs may promote the spread of infection, especially in the treatment of acne [140]. Therefore, more long-term and in-depth in vivo safety assessment studies are needed. There are difficulties in precisely controlling drug loading and release. Different skin diseases have different requirements for drug dosage, release rate, and time for treatment [141–143]. Currently, MNs technology has difficulty in meeting these individualized needs fully, which can easily lead to premature, rapid, or incomplete drug release, adversely affecting the treatment efficacy and potentially increasing the risk of drug toxicity. The manufacturing process of MNs is complex and costly. High-precision MNs manufacturing requires advanced equipment and techniques, and quality control during the production process is difficult, resulting in the high price of some MNs products [144–146], limiting their wide application in clinical practice. Although the emergence of artificial intelligence and sensing technologies allows for MNs with the functions of diagnosing, monitoring, and treating diseases, they are still in the initial stage of development and face many challenges [147, 148]. The system used for evaluation of clinical efficacy is not yet perfect. Currently, there is a lack of unified and standard efficacy evaluation indicators and methods for MNs treatment of skin diseases. Different studies often use different evaluation parameters, making it difficult to objectively and accurately compare and comprehensively analyze the treatment effects of MNs, hindering the standardized promotion of MNs technology. Despite the challenges faced by MNs clinical applications, with the continuous development and cross-integration of multiple disciplines such as materials science, medical engineering, and pharmacy, the prospects of MNs technology in the field of the treatment of dermatological diseases remain bright. Therefore, in future, more attention could be paid to: developing new MNs materials with good biocompatibility and intelligent response; optimizing drug loading and precise delivery techniques to achieve targeted and sustained release drug delivery; developing new high-precision MNs manufacturing techniques, such as the combination of 3D printing, micro-nano processing, and self-assembly technologies; promoting the integration of artificial intelligence and microelectronic technology to achieve personalized treatment with MNs; exploring the synergistic mechanisms associated with the combination of MNs with other therapies; establishing a unified and objective efficacy evaluation standard system, etc., to enhance the precision, effectiveness, and standardization of MNs treatment.

In conclusion, further research is necessary to explore the development of new approaches for the more efficient integration of bioactive compounds into MN structures, thereby facilitating the more widespread clinical application of these drug delivery techniques. In recent years, the research on MNs in disease diagnosis has attracted greater scientific attention, as they enable the collection of biological samples with minimal pain and are expected to provide a more comfortable and convenient method for clinical diagnosis. Similarly, MNs can be integrated with sensor technology to monitor a variety of physiological indicators in real-time, providing powerful data support for personalized medicine. Despite the significant progress that MNs-based drug delivery platforms have made to date, their translational use will require further advancements. The safety and stability of MNs are key considerations in their clinical translation, making them the main concerns of clinical research on MNs. Therefore, research that concentrates on the application of MNs in the management of skin diseases remains a major clinical interest. Finally, through continuous research innovation and technological improvement, MNs are expected to become an important means of treating skin diseases, bringing safer, more efficient and personalized treatment options to a large number of patients.
